# PRL2 inhibition elevates PTEN protein and ameliorates progression of acute myeloid leukemia

**DOI:** 10.1172/jci.insight.170065

**Published:** 2023-10-09

**Authors:** Colin Carlock, Yunpeng Bai, Allison Paige-Hood, Qinglin Li, Frederick Nguele Meke, Zhong-Yin Zhang

**Affiliations:** 1 Department of Biochemistry,; 2 Department of Medicinal Chemistry and Molecular Pharmacology,; 3 Department of Biological Sciences,; 4 Department of Chemistry,; 5 Institute for Cancer Research, and; 6 Institute for Drug Discovery, Purdue University, West Lafayette, Indiana, USA.

**Keywords:** Hematology, Therapeutics, Leukemias, Phosphoprotein phosphatases, Tumor suppressors

## Abstract

Overexpression of phosphatases of regenerating liver 2 (PRL2), detected in numerous diverse cancers, is often associated with increased severity and poor patient prognosis. PRL2-catalyzed tyrosine dephosphorylation of the tumor suppressor PTEN results in increased PTEN degradation and has been identified as a mechanism underlying PRL2 oncogenic activity. Overexpression of PRL2, coincident with reduced PTEN protein, is frequently observed in patients with acute myeloid leukemia (AML). In the current study, a PTEN-knockdown AML animal model was generated to assess the effect of conditional PRL2 inhibition on the level of PTEN protein and the development and progression of AML. Inhibition of PRL2 resulted in a significant increase in median animal survival, from 40 weeks to greater than 60 weeks. The prolonged survival reflected delayed expansion of aberrantly differentiated hematopoietic stem cells into leukemia blasts, resulting in extended time required for clinically relevant leukemia blast accumulation in the BM niche. Leukemia blast suppression following PRL2 inhibition was correlated with an increase in PTEN and downregulation of AKT/mTOR-regulated pathways. These observations directly established, in a disease model, the viability of PRL2 inhibition as a therapeutic strategy for improving clinical outcomes in AML and potentially other PTEN-deficient cancers by slowing cancer progression.

## Graphical abstract

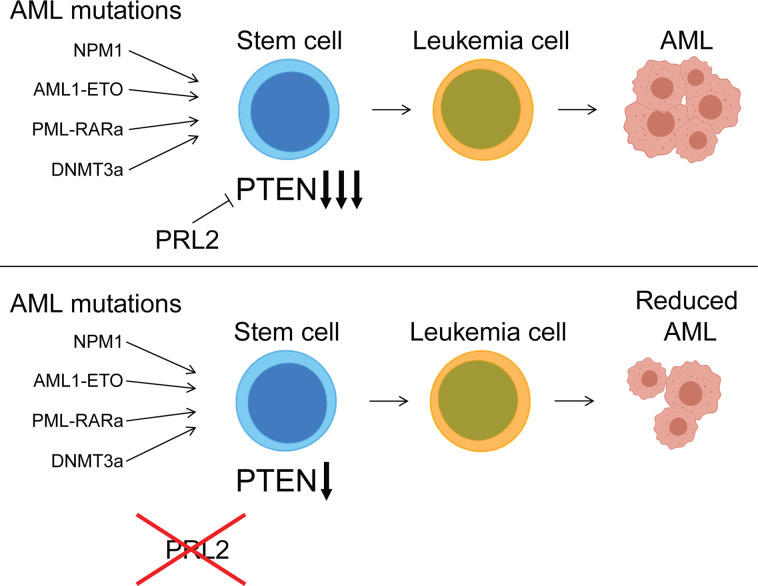

## Introduction

Precise regulation of protein tyrosine phosphorylation is essential for maintaining cellular homeostasis. The importance of protein tyrosine kinases (PTKs) in regulating cellular signaling is well established; however, the role of protein tyrosine phosphatases (PTPs) in reversing or blocking kinase-activated signaling cascades is not yet fully appreciated (1). Increasing evidence supports a critical role of PTPs in the pathogenesis of many diseases, such as the identification of numerous PTPs as potential oncogenes and tumor suppressors (2). Understanding the functional role of PTPs in disease has been hampered by poorly defined PTP substrate specificity and a lack of identified biological targets.

The phosphatases of regenerating liver (PRL) is a 3-member PTP subfamily, composed of PTP4A1 (PRL1), PTP4A2 (PRL2), and PTP4A3 (PRL3), that shares substantial amino acid sequence homology (3). Overexpression of the PRLs has been associated with many types of human cancer and is frequently prognostic of worsened patient outcomes (4–6). However, the mechanisms by which the PRLs contribute to tumorigenesis are poorly understood, and both catalytic and noncatalytic roles for PRLs have been proposed (5, 7). Recently, the tumor suppressor protein PTEN was identified as a PRL2 substrate (7). PTEN is frequently downregulated in cancer, resulting in activation of the PI3K/AKT/mTOR pathway and consequent enhanced cell growth and proliferation (8). PRL2 was demonstrated to dephosphorylate PTEN at the Y336 residue, resulting in increased NEDD4-mediated polyubiquitination and subsequent proteasomal degradation of PTEN (7). Furthermore, whereas PTEN heterozygous mice spontaneously developed tumors, concomitant deletion of PRL2 led to an increased abundance of PTEN protein, decreased AKT phosphorylation, reduced activity of the P13K/AKT/mTOR pathway, and decreased tumor formation (7). The ability of PRL2 to downregulate PTEN and promote AKT activation provides a plausible mechanism for PRL2-mediated oncogenesis. However, the studies utilized a constitutive model, of both PRL2 deletion and PTEN deficiency, which may not accurately recapitulate the complex regulatory roles of PTEN and PRL2 during development and in tumor suppression. The impact of PRL2 modulation of PTEN expression on pathogenesis and tumorigenesis within a specific cancer type, therefore, remains to be demonstrated.

Acute myeloid leukemia (AML) is the most lethal form of leukemia, responsible for more than 147,000 reported deaths worldwide in 2019 (9, 10). AML is a blood cancer characterized by the transition of hematopoietic stem cells (HSCs) to leukemia stem cells (LSCs) within the BM as a result of ectopic mitotic entry and altered differentiation pathways (11). Expansion of LSCs and the consequent decrease in mature myeloid cell populations clinically manifests as bleeding, anemia, or severe infections. AML is a multifaceted disease, and patients most often present with complex karyotypes composed of 3 or more mutations from an array of discrete mutation categories (12, 13). To date, this underlying mutational complexity has hindered the development of therapies capable of targeting the variety of mutations in AML while maintaining broad applicability to the patient populations. However, one common feature observed in AML clinical samples is a significant reduction in the relative amount of PTEN protein level in HSCs (14, 15). The observed deficiency in PTEN protein and function in HSCs is consistent with the proposed role of PTEN as a negative regulator of LSC proliferation and is coincident with the HSC-to-LSC transition observed in AML (16, 17). In contrast to many other cancers where PTEN loss of function is driven by PTEN gene mutations, there is a paucity of PTEN genetic mutations in AML. Instead, PTEN protein deficiency is reflective of aberrant posttranslational modifications that arise from alterations in PTEN interacting proteins, leading to PTEN inactivation and degradation (14, 18, 19). Restoration of PTEN protein level could, therefore, represent a broadly applicable therapeutic strategy in AML. In addition, it has also been observed that PRL2 is frequently overexpressed in patients with AML, and such overexpression is associated with more severe clinical outcome (20–23). Analogous to PTEN, PRL2 mutations are rarely if ever detected in patients with AML (24). Given the inverse correlation of PRL2 overexpression and the reduction in PTEN protein level in clinical samples of AML, together with the role of PTEN in the regulation of HSC proliferation (17), an AML mouse model was generated to further investigate the regulatory role of PRL2 on PTEN protein level, PTEN-regulated pathways, and the onset and progression of the AML phenotype. This AML model provided for conditional, adult-onset PTEN heterozygosity and PRL2 deletion.

Animals in which PRL2 was conditionally deleted exhibited survival curves, cellular phenotypes, and AKT/mTORC1 pathway activity similar to that of WT control mice. The PTEN heterozygote (PTEN HET) animals had significantly shorter survival times, and the majority of the animals displayed characteristics consistent with AML at the time of death. In contrast, deletion of PRL2 in the PTEN HET mice resulted in an approximately 30% increase in the amount of PTEN protein and a concomitant reduction in AKT pathway activation, a slower rate of LSC accumulation within the BM, and a delayed onset of severe leukemia phenotypes relative to the PTEN HET counterparts. The observations support the hypothesis that PRL2 deletion reduces PTEN turnover and significantly delays the onset of leukemia phenotype resulting from decreased levels of PTEN protein. Restoration of PTEN protein levels through PRL2 inhibition may therefore represent an alternative therapeutic approach for treatment in delaying progression of AML. In addition, given the ubiquity of PTEN inactivation through downregulation or degradation in numerous cancer types (25), inhibition of PRL2 may be applicable not only to AML but also to a potentially broad range of cancers.

## Results

### Generation of a PRL2 conditional transgenic mouse for investigating PRL2 function.

Previous studies have utilized a conventional PRL2–constitutive KO mouse model to demonstrate that PRL2 deletion delays PTEN heterozygosity–induced spontaneous tumorigenesis (7). In order to further explore PRL2 functionality, with greater temporal control of PRL2 expression, a conditional PRL2 mouse was generated using CRISPR/Cas9 that contains flanking loxP sites enclosing *Prl2* exon 4 (Supplemental Figure 1A; supplemental material available online with this article; https://doi.org/10.1172/jci.insight.170065DS1). PCR genotyping confirmed the generation of founder mice with loxP insertion in both intron 3 and 4 (Supplemental Figure 1B). After crossing the *Prl2*-floxed founder with PRL2 WT mice, homozygous *Prl2*^fl/fl^ mice were obtained (Supplemental Figure 1, C and D). The functionality of the *Prl2*^fl/fl^ allele mice was then validated by breeding them with mice possessing a Rosa26-CreER^T2^ transgene and inducing Cre recombination, whereupon loss of PRL2 protein and an increase in PTEN was observed (Supplemental Figure 1E).

### Generation of a conditional PTEN knockdown, radiation-induced leukemia mouse model for in vivo AML analyses.

A functional consequence of many leukemogenic mutations in patients with AML is an appreciable reduction in PTEN protein abundance (14, 18, 19). Deletion of one or both PTEN alleles has also been previously shown to induce development of LSCs capable of leading to an AML-like phenotype in vivo (26, 27). Despite these observations, few AML model systems have provided a platform to interrogate either the effect of changes in PTEN protein level on disease development or the potential of modulating PTEN as a therapeutic strategy (28, 29). Previous models based on a single mutation have been hindered by latency and penetrance of the disease phenotype, while the generation of models containing multiple mutations is technically demanding and requires exceedingly stringent analyses and data interpretation (30). Furthermore, while a number of specific mutations have been implicated in modulating PTEN in AML (e.g., PML-RARa, NPM1c, and CSK2), the effect on PTEN activity and PTEN protein levels in models utilizing these mutations is not well understood. Models that inactivate PTEN through point or missense mutations would also be inappropriate, since patients with AML do not exhibit PTEN genetic mutations. Constitutive PTEN heterozygous animals have numerous confounding variables due to changes during development, and conditional PTEN deletions across numerous cancer models have shown substantial disease latency (27, 31). Systems that relied on full PTEN KO in stem cell populations resulted in early-onset myeloproliferative disorders that predominantly develop into T-ALL phenotype with minor AML development (32). To overcome these limitations, a conditional PTEN HET and PRL2-KO;PTEN HET irradiated mouse model system was developed.

The conditional animal model exhibiting an AML phenotype was generated by cross-breeding mice possessing PTEN-floxed alleles with mice carrying the conditionally activated Rosa26-Cre-ER^T2^ promoter to obtain Rosa26^Cre/Cre^ WT and the inducible Rosa26^Cre/Cre^;PTEN^wt/fl^ PTEN heterozygous (PTEN HET) mice. Prior studies using PTEN heterozygous animals noted considerable delay in leukemia onset compared with PTEN-KO animals (31). The latency was attributed to the residual activity of the remaining PTEN allele to respond to moderate levels of DNA damage over time. In order to enhance leukemia development in the current study, a sublethal dose of ionizing radiation was used to rapidly introduce widespread, nonspecific, DNA lesions. Radiation treatment, and the resulting induction of mutations that enhance LSC generation, is a well-established method of generating leukemia in animal models (29, 33). Several protocols were developed in order to optimize the timely induction of AML in 10-week-old PTEN HET mice with the fewest detectable off-target phenotypes (Supplemental Figure 2A). Since AML does not generate a solid tumor, weight change was used as a proxy measurement of AML progression and overall animal health (Supplemental Figure 2B). Each of the tested induction protocols was capable of inducing leukemia-like phenotypes within a 20- to 40-week time frame, similar to previously published single-mutant, AML mouse models (34, 35) (Supplemental Figure 2C).

### Generation of PRL2-KO;PTEN HET mice.

**Figure 1:**
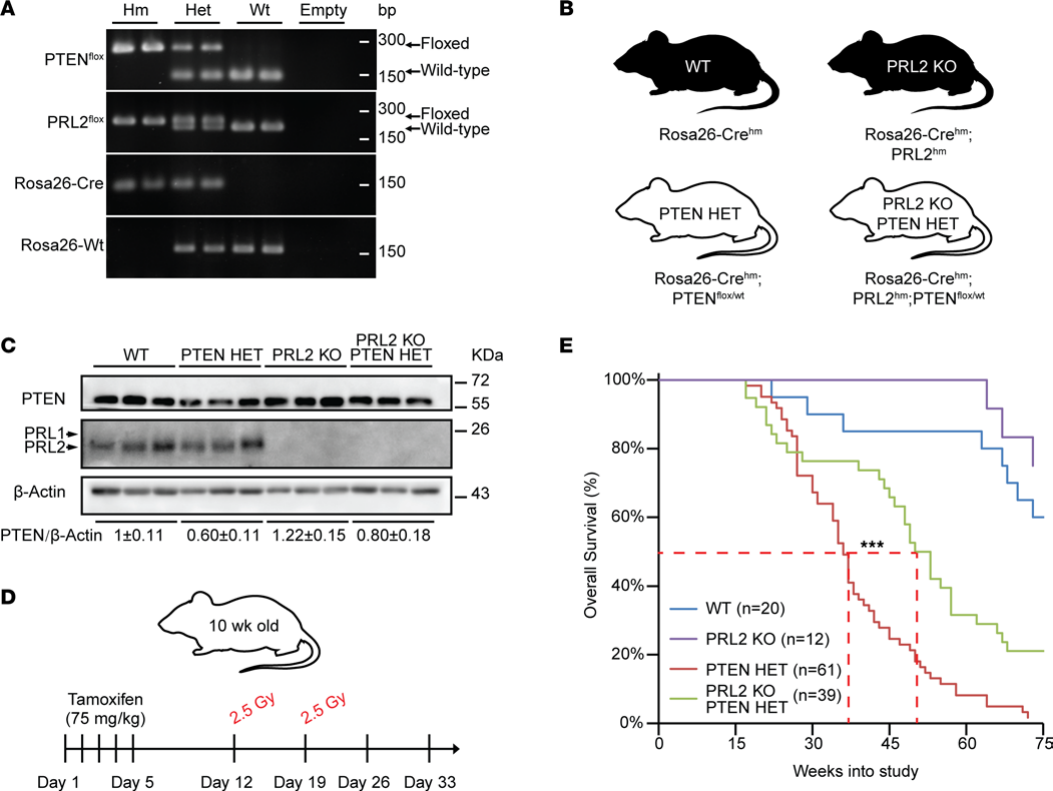
PRL2 deletion greatly enhances overall survival in the PTEN HET radiation-induced leukemia mouse model. (**A**) PCR genotyping demonstrating discrimination of the homozygous (Hm), heterozygous (Het), and WT alleles for PTEN, PRL2, and Rosa-Cre transgenes. (**B**) Genotypes of the transgenic mouse groups used for the study. (**C**) Western blot analysis of bone marrow lysates from transgenic mouse groups. Average relative abundance of PTEN in each animal group is given. Sample size of *n* = 9 for each animal group. (**D**) Treatment protocol used for inducing AML in animal models (Gy = gray, absorbed dose of radiation). (**E**) Kaplan-Meier survival plot of overall survival in mouse groups after AML induction. Median survival time indicated with dashed lines. ***P < 0.00002. Statistical significance was calculated using Kaplan-Meier log-rank test.

With an AML induction protocol identified, mice possessing the PRL2-floxed transgene were then bred with the WT and PTEN HET mice. Presence of the PTEN and PRL2 transgenes was validated through genotyping (Figure 1A), and mice were segregated into WT, PRL2-KO, PTEN HET, or PRL2-KO;PTEN HET experimental groups (Figure 1B). Transgene functionality within the 4 groups was validated by measuring the relative protein expression of PTEN and PRL2 in the BM following tamoxifen-induced recombination (Figure 1C). PTEN HET BM samples exhibited the expected ~50% decrease in PTEN compared with WT mice. No appreciable PRL2 protein was detected in either the PRL2-KO or PRL2-KO;PTEN HET cohorts. In addition, the PRL2-KO animals demonstrated a relative 20%–30% increase in PTEN protein (WT versus PRL2-KO and PTEN HET versus PTEN HET;PRL2-KO), consistent with previous in vivo PRL2-KO studies and the proposed role of PRL2 in catalyzing PTEN turnover (7, 36).

### PRL2 deletion significantly improves survival in PTEN HET transgenic mice.

Equal numbers of male and female mice from control and experimental groups were treated using the optimized AML induction protocol (Figure 1D). Weight was used as a proxy measure of animal health and disease progression, while disease pathogenesis was determined by necropsy at time of death. Over the course of the study, WT and PRL2-KO groups exhibited similar survival profiles, with a median survival time of more than 75 weeks after induction (Figure 1E). The PTEN HET cohort by comparison displayed a statistically significant decrease in survival, with a median survival time of 36 weeks after induction. In contrast, the simultaneous deletion of PRL2 in the PTEN HET animals (PRL2-KO;PTEN HET) resulted in an extension of median survival time from 36 weeks to 50 weeks after induction, indicating that PRL2 inhibition in a PTEN-deficient system has a positive effect on animal survival. No significant differences were observed between male and female cohort survival in any of these experimental groups.

Given the role of PTEN as a tumor suppressor, and corresponding increased cancer susceptibility when PTEN protein levels are reduced, animals were characterized at time of death for the incidence of specific cancer phenotypes. There was no significant incidence of cancer in the WT and PRL2-KO animals at 36 weeks after induction (Supplemental Table 1). The PTEN HET cohort exhibited a significantly increased cancer incidence, with the predominant phenotype consistent with that of AML. Such a phenotype was not significant in the PRL2-KO;PTEN HET animals, implying a role for PRL2 inhibition in the reduction of AML phenotypes in these animals and a possible explanation for the increase in survival time.

It is also notable that the mortality rates for PTEN HET and PRL2-KO;PTEN HET groups were comparable during the earlier (20- to 27-week) postinduction time period (Figure 1E). Within this time frame, a plurality of PTEN HET, and the entirety of PRL2-KO;PTEN HET, animal deaths could be attributed to the development of an ALL phenotype, as evidenced by an enlarged thymus and lack of other cancer-related phenotypes during necropsy. In addition, analyses of these thymus tissues indicated that PTEN loss of heterozygosity (LOH) was prevalent in samples from both groups (our unpublished observations). ALL is a characteristic outcome of complete PTEN loss within HSC populations (32). Outside of this window, the incidence rate of ALL or PTEN LOH in PTEN HET and PRL2-KO;PTEN HET animal groups decreased significantly. Additionally, none of the animals that died of ALL appeared to have any characteristics of AML development. As previously noted above, no significant differences in incidence rates between male and female animals was observed. These observations further support the integrity of the current PTEN HET conditional model in recapitulating the leukemic phenotype previously reported in PTEN-null or -deficient animals.

### PRL2 deletion upregulates PTEN and reduces AKT/mTOR/S6K signaling, resulting in reduced proliferation.

A hallmark of numerous cancers is a reduction in PTEN resulting in increased phosphorylation of AKT and activation of AKT/mTOR-regulated pathways (8, 37). Increased activity of the AKT/mTOR pathway has been reported in more than 60% of patients with AML and is associated with decreased patient survival (38). The AKT/mTOR signaling axis regulates multiple downstream processes that reflect a complex signaling network with interactions that have been implicated in hematopoiesis, leukemogenesis, and the activity of other cells within the BM niche (38). The effect of PRL2 deletion on PTEN protein level and downstream AKT pathways, including those involved in proliferation and apoptosis, were examined in the current animal model system.

**Figure 2:**
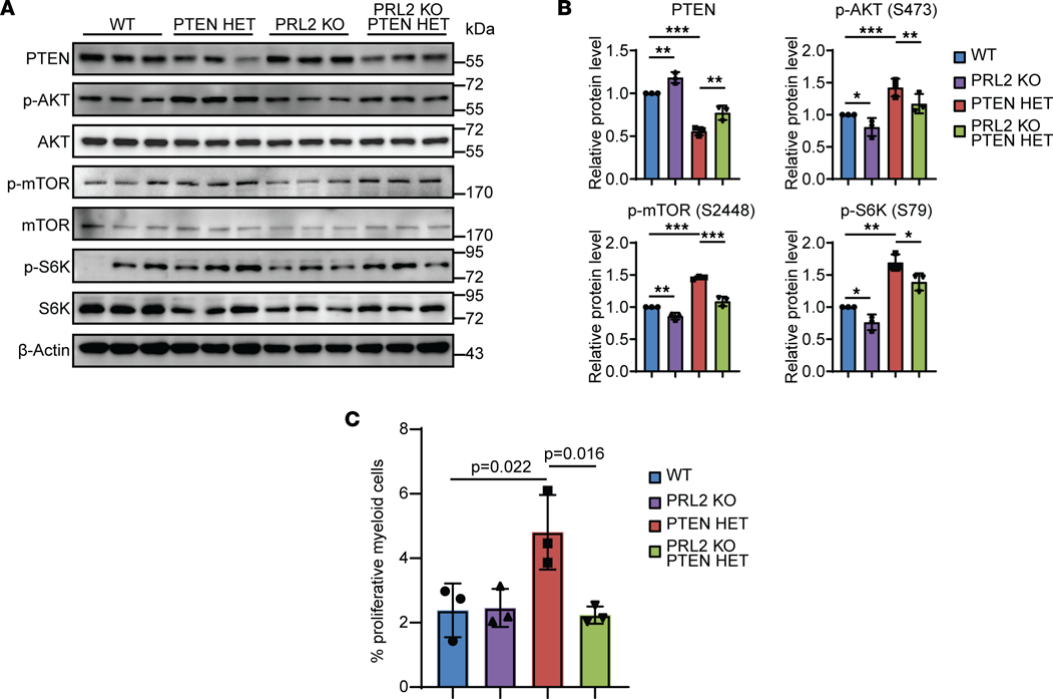
PRL2 deletion upgregulates PTEN activity to reduce AKT pathway signaling and reduce proliferation in the PTEN HET leukemia model. (**A**) Western blot analysis of PTEN and AKT pathway components in 1-week postinduction spleen samples from transgenic mouse groups. (**B**) Quantification of PTEN and AKT pathway protein levels from **A**; error bars represent 3 independent experiments. *P < 0.05, **P < 0.02, ***P < 0.001. (**C**) Quantification of proliferation seen in myeloid cell populations (CD11b^+^, CD11c^+^, and GR-1^+^) of 1-week spleens with a given *P* value. Sample size of *n* = 3 for each animal group. Statistical significance was calculated using 1-way ANOVA with a post hoc Tukey’s HSD test.

Protein was isolated from splenic myeloid cells 1 week following tamoxifen induction but prior to irradiation, in order to establish the baseline protein activity within the relevant pathways prior to the appearance of random, radiation-induced genetic lesions (Figure 2A). Splenic populations, rather than BM HSCs, were assayed in consideration of the large number of quiescent HSCs in BM that could skew the pathway analysis. Western blot analysis revealed that, in comparison with WT samples, there was an average 18% increase in relative PTEN abundance in PRL2-KO animals, in accordance with previously published models of PRL2 deletion (7, 36) (Figure 2B). The PTEN HET animals exhibited an average decrease of 44% in PTEN abundance compared with WT. In contrast, PRL2-KO;PTEN HET animals displayed a statistically significant relative increase in PTEN protein abundance of 22% over the PTEN HET cohort. Consistent with the known role of PTEN in downregulating AKT signaling, the relative AKT phosphorylation in the experimental groups reflected the observed PTEN abundance. The PRL2-KO mice demonstrated a 14% decrease in AKT phosphorylation relative to WT animals, whereas there was an average 70% increase in AKT phosphorylation in the PTEN HET mice. The effect of reduced PTEN expression on AKT phosphorylation was attenuated in the corresponding PRL2-KO;PTEN HET mice, where a 30% decrease in relative AKT phosphorylation was detected. Similar trends were observed in the phosphorylation status of the downstream AKT target, mTOR, which, when activated by phosphorylation, promotes pathways involved in protein synthesis and cell growth. Relative to WT, the PRL2-KO mice exhibited a 14% reduction in mTOR phosphorylation, whereas the PTEN HET mice demonstrated a 46% increase in mTOR phosphorylation. In a similar trend to phospho-AKT, the PRL2-KO;PTEN HET animals displayed a 37% decrease in mTOR phosphorylation relative to PTEN HET mice. While there are a number of downstream targets of mTORC1, the ribosomal protein S6K is of particular importance in AML, having been shown to be involved in leukemogenesis and LSC self-renewal (39). Compared with WT, the PRL2-KO animals demonstrated a 19% drop in S6K phosphorylation, consistent with that observed for AKT and mTOR phosphorylation. A relative increase in S6K phosphorylation of 42% was detected in PTEN HET animals compared with WT. The PRL2-KO;PTEN HET mice, by comparison, exhibited a 25% decrease in S6K phosphorylation, compared with the PTEN HET animals. One of the most established functions of AKT/mTOR/S6K signaling is the regulation of cell proliferation through control of cell cycle progression. The physiological impact of the changes to AKT signaling in the PTEN HET and PRL2-KO;PTEN HET splenic populations was assessed by measuring the amount of proliferation within myeloid populations (Figure 2C). The relative percentage of proliferative CD11b, GR-1, and CD11c myeloid populations was calculated and combined for each animal group. In WT and PRL2-KO animals, approximately 2.5% of cells within these myeloid populations were proliferative. In PTEN HET animals, proliferation increased to 4.8%, in accordance with the expected phenotype based on the changes in AKT signaling. The PRL2-KO;PTEN HET animals, however, displayed a relative proliferation comparable with WT and PRL2 KO at only 2.2%. These observations suggest that, while the level of AKT activation in PRL2-KO;PTEN HET samples was somewhat elevated compared with WT and PRL2 KO, the extent of AKT activation was insufficient to impact a proliferative phenotype at this time point.

In addition to regulating proliferation, AKT signaling also controls cell survival through the suppression of apoptosis. To more broadly investigate the impact of AKT signaling changes in this model system, markers of apoptosis were analyzed (Supplemental Figure 3A). Direct antiapoptotic substrates of AKT, phospho-MDM2, and phospho-BAD, were analyzed, in addition to general markers of apoptotic progression such as Caspase 3, cleaved Caspase 3, Caspase 9, PARP, and cleaved PARP. Few consistent statistically significant changes were detected in any of these markers across any group with the exception of Caspase 3 and phospho-BAD (Supplemental Figure 3B). The physiological importance of these changes appears negligible, since total apoptosis in CD11b, GR-1, and CD11c populations as assessed by TUNEL assay revealed no statistically significant changes in apoptosis between the WT and experimental animal groups (Supplemental Figure 3C). Furthermore, examination of PTEN phosphorylation revealed an approximately 20% increase in PTEN Y336 phosphorylation in PRL2-KO groups, consistent with the proposed PRL2 mechanism of PRL2-regulated PTEN dephosphorylation (7) (Supplemental Figure 3, D and E).

In the current AML model system, the reduced PTEN protein is associated with increased activation of AKT/mTOR/S6K signaling and proliferation, potentially contributing to a predisposition toward leukemogenesis and reduced survival of the PTEN HET mice. PRL2 deletion increased the abundance of PTEN protein and attenuated the proliferative phenotype, providing an explanation for the significantly improved survival, which is suggestive of reduced AML pathogenesis in the PRL2-KO;PTEN HET animals.

### PRL2 deletion impairs the expansion of LSC progenitor populations in the BM of PTEN HET mice.

**Figure 3:**
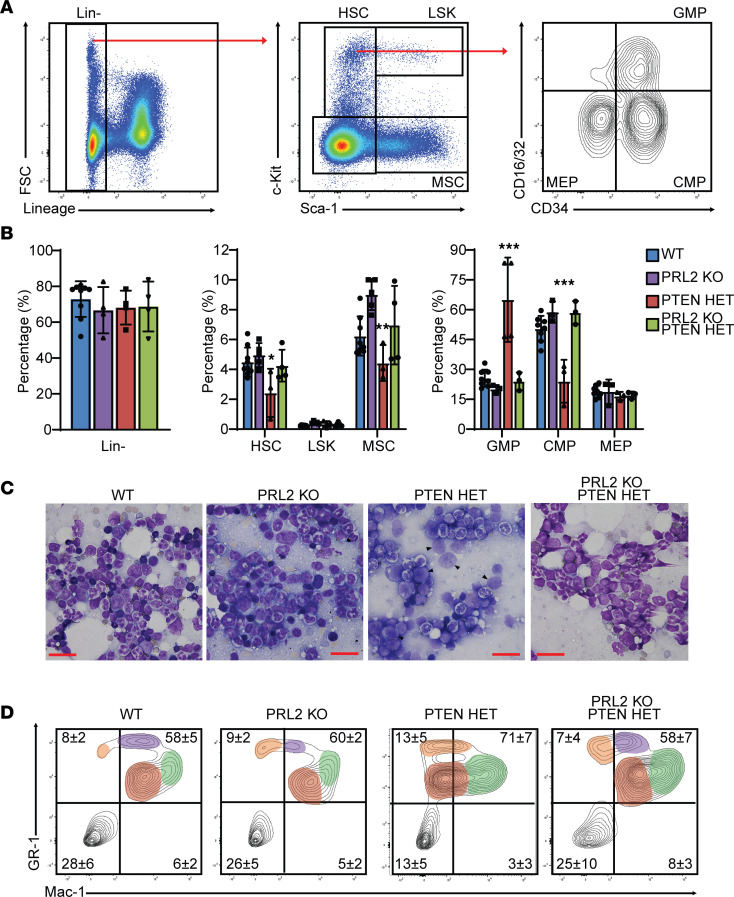
PRL2 deletion reduces AML phenotype burden in bone marrow populations. (**A**) Example gating strategy for FACS analysis of Lineage, Sca-1, and c-Kit sorted stem cell progenitor populations isolated from WT bone marrow. (**B**) Relative abundance of bone marrow subpopulations isolated from 40-week mice as a percentage of the total subpopulation. Samples sizes for each group: WT, *n* = 8; PTEN HET, *n* = 4; PRL2 KO, *n* = 4; PRL2-KO;PTEN HET, *n* = 4. Significance calculated relative to WT. ***P < 0.001. (**C**) Representative images of bone marrow cell morphology in 40-week mouse groups with blast-like cells and clusters indicated with arrowheads. Visualized with Wright-Giemsa stain. Scale bars: 25 μm. (**D**) Representative FACS analysis of residential mature myeloid cell markers in 40-week bone marrow. Unique population clusters highlighted with red, green, orange, or purple. Relative abundance of populations quantified for each quadrant based on sample sizes of WT, *n* = 10; PTEN HET, *n* = 8; PRL2 KO, *n* = 4; PRL2-KO;PTEN HET, *n* = 8. Statistical significance was calculated using 1-way ANOVA with a post hoc Tukey’s HSD test.

AML arises from complex, cooperating mutations in the HSC populations of the BM, resulting in abnormal proliferation or differentiation of HSC-derived myeloid cells leading to the downstream development of LSCs and eventually leukemia blasts (40). BM populations from age-matched 40-week postinduction animals from the WT and experimental groups were compared in order to investigate possible mechanisms by which PRL2 deletion improved the survival of the PTEN HET mice. Consistent with the necropsy data, there were no consequential changes in the lymphoid lineage populations to indicate ALL as a predominant phenotype at this time point (Supplemental Figure 4A). Myeloid lineage populations were then examined using established markers (41). The markers and gating strategy were chosen to distinguish major hematopoietic subpopulations, including Lineage^–^Sca1^–^c-Kit^+^ (Lin^–^Sca1^–^c-Kit^+^) HSC populations, Lin^–^Sca1^+^c-Kit^+^ (LSK) stem cell and multipotent progenitor populations, as well as Lin^–^Sca1^+^c-Kit^–^ mesenchymal stromal cells (MSC). Within the HSC group, hematopoietic precursor populations were categorized as CD16/32^–^CD34^–^ megakaryocyte-erythroid progenitor (MEP), CD16/32^–^CD34^+^ common myeloid progenitor (CMP), and CD16/32^+^CD34^+^ granulocyte-macrophage progenitor (GMP) cells (Figure 3A). Comparison between the cohorts of the Lin^–^ cells revealed no statistically significant changes in the proportion of Lin^–^ cells between the 40-week mouse groups (Figure 3B). Within the Lin^–^ populations, no difference was observed in the relative abundance of HSC and LSK cell populations between WT and PRL2-KO animals, though the abundance of MSCs within PRL2-KO animals was significantly elevated in a manner not seen in any other treatment groups. In contrast to the WT and PRL2-KO mice, PTEN HET mice displayed a significant decrease in relative abundance of cells identifying as HSCs. This observation is consistent with observation reported for other AML models, where PTEN loss has been attributed to LSC-inducing mutations (16), as well models where upregulated AKT/mTOR has contributed to depletion of HSC pools through purported changes to proliferation, differentiation, and survival (38). Importantly, the decrease in HSCs is not apparent in the PRL2-KO;PTEN HET animals, which exhibited a relative HSC abundance that resembled the WT and PRL2 KO groups. These observations suggest that the increase in PTEN and reduction in AKT/mTOR signaling arising from PRL2 deletion may be sufficient to significantly impair the loss in HSC populations that arose from the induced PTEN deficiency.

Abnormal HSC differentiation or proliferation is amplified throughout hematopoiesis and strongly contributes to the accumulation of LSCs and AML (42). To better understand the downstream implication of the HSC decrease in PTEN HET mice, myeloid precursor populations (MPP) within the HSC niche were further analyzed to detect any developmental or differentiation abnormalities. Comparison of the MPPs within the HSC niche of each mouse group revealed a similar trend in relative population abundance of GMP (WT, 26%; PRL2 KO, 20%), CMP (WT, 50%; PRL2 KO, 59%), and MEP (WT, 19%; PRL2 KO, 19%) cells between WT and PRL2-KO mouse groups that appeared indicative of healthy hematopoiesis. The PTEN HET mice, however, demonstrated a significant increase in the relative concentration of GMP cells, with an average abundance of 59%. This increase in GMP concentration led to a corresponding decrease in CMP cells, averaging 24% of the population. MEP abundance in PTEN HET MPPs appeared relatively unchanged compared with WT or PRL2 KO. Expansion of GMP cells e correlated to impaired stem cell differentiation, and can result in the generation of LSCs, leukemia blasts, and the development of AML. AKT phosphorylation has a noted role in regulating differentiation and proliferation of HSCs through control of HSC quiescence, and enhanced AKT phosphorylation as a result of reduced PTEN abundance could be a contributing factor to the observed GMP expansion phenotype (43). Deletion of PRL2 in the PRL2-KO;PTEN HET animals was able to overcome this phenotype, displaying relative abundances of GMP (24%), CMP (59%), and MEP (17%) cells that were statistically similar to the healthier WT and PRL2-KO mouse groups. This further supports the earlier observation of HSC abundance, which indicates that the increased level of PTEN protein and decreased AKT activity in the PRL2-KO;PTEN HET animals is able to attenuate the leukemia-like phenotype seen in PTEN HET alone and restore a phenotype more closely related to a WT state.

In addition to altered differentiation, aberrant proliferation is a hallmark of AML and is regulated by AKT/mTOR signaling in a variety of cancers (38). While the impaired hematopoiesis seen in the PTEN HET mice would be capable of generating LSCs, it needed to be determined whether these LSC populations could proliferate sufficiently to induce a full AML phenotype. To assess LSC and leukemia blast abundance, morphological characterization was performed on BM aspirates from 40-week postinduction animals (Figure 3C). LSCs and AML progression can be identified in the BM by an abundance of leukemia blast–like cells with a high nuclear/cytoplasm ratio, poorly defined or disordered chromatin, or unusual nuclear lobulation. Both WT and PRL2-KO animal groups were devoid of noticeable leukemia blast development and exhibited a wide variety of developing cell morphologies indicative of healthy hematopoiesis. PTEN HET mice often exhibited a > 20% leukemia blast count, consistent with the International Prognostic Scoring System (IPSS) metrics used clinically to define severe AML in patients and indicating that appreciable proliferation of leukemia blasts in the BM did occur in this model. Consistent with the HSC population data, deletion of PRL2 and elevation of PTEN protein in the PRL2-KO;PTEN HET animals considerably reduced leukemia blast count and restored a more WT phenotype. The downstream impact of leukemia blast accumulation and proliferation was assessed by analyzing the composition of mature myeloid populations residing within the BM. Using the established myeloid markers GR-1 and Mac-1, discrete mature myeloid populations within the BM could be identified (Figure 3D). The WT and PRL2-KO mice exhibited several distinct myeloid population clusters expressing various marker abundances (GR-1^lo^, GR-1^med^, GR1-^hi^, Mac-1^lo^, Mac-1^hi^), consistent with typical hematopoietic development and as seen in the diverse of cell morphologies observed with microscopy. Within PTEN HET samples, the distinct population clusters were reduced to more homogenized groups of GR-1^+^ cells expressing a gradient of Mac-1. Such population homogenization is a hallmark of impaired differentiation capabilities characteristic of leukemia blasts (44) and is consistent with the development of AML reported for other mouse models of AML. In contrast, PRL2-KO;PTEN HET mice did not exhibit this population homogenization and, instead, showed the distinct population clusters observed in WT and PRL2 KO indicative of normal hematopoiesis.

Comprehensive analysis of the BM indicates that the PTEN HET mice displayed changes in their HSC and MPP populations characteristic of an AML-like phenotype arising from impaired progenitor cell differentiation, leading to the generation of LSC and leukemia blast populations that results in the disruption of normal residential myeloid populations. The underlying changes in HSC and MPP identity can be correlated to the decrease in the PTEN protein level and suggest that the subsequent observed increase in PI3K/AKT/mTOR pathway activity provided an environment preconditioned toward enhanced proliferation and cellular activity for the cooperating irradiation-induced mutations. The observed PTEN deficiency phenotype was rescued by the increase in PTEN protein level as evidenced by the PRL2-KO;PTEN HET mice (Figure 3, B and D).

### PRL2 deletion reduces leukemia blast presentation in circulating blood.

**Figure 4:**
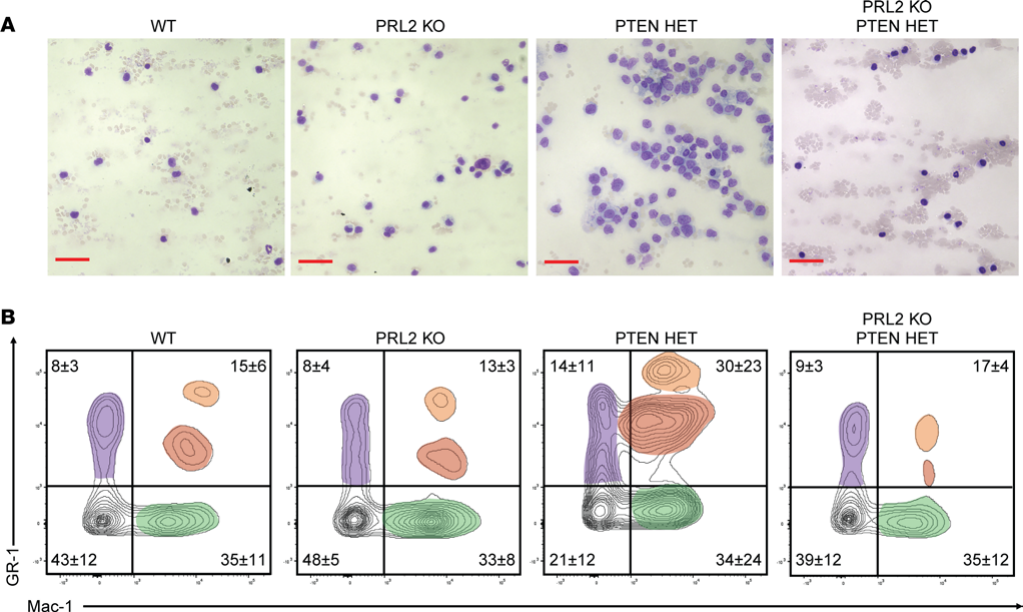
Myeloid leukocyte populations in the circulating blood of PTEN HET mice demonstrate AML-like population changes, with PRL2 deletion significantly reducing leukocyte burden. (**A**) Representative microscopy of peripheral blood smear from a 40-week mouse. Leukocyte nuclei stain (purple), cytoplasm (blue), and RBCs (pale pink) are shown. Visualized with Wright-Giemsa stain. Scale bars: 50 μm. (**B**) Representative FACS analysis of whole blood from age-matched 40-week mice. Unique population clusters highlighted with red, green, or purple. Relative abundance of populations quantified for each quadrant based on sample sizes of WT, *n* = 13; PTEN HET, *n* = 5; PRL2 KO, *n* = 5; PRL2-KO;PTEN HET, *n* = 5.

The BM HSC analyses reflect a phenotype consistent with AML in the 40-week postinduction PTEN HET animals that was not apparent in the other cohorts. To further validate the development of AML in the conditional PTEN HET AML model, and the reduction in disease development as a result of PRL2 inhibition, additional tissues were analyzed for the presence of AML-associated phenotypes. AML progression and increased severity was characterized by the appearance of high concentrations of leukemia blast cells within the circulating blood. Circulating leukocytes from the blood of age-matched 40-week animals from each mouse group were analyzed for changes in abundance and morphology (Figure 4A). Leukocytes from WT and PRL2-KO mice exhibited diverse nuclear morphology characteristic of normal hematopoiesis. In contrast, blood samples from the PTEN HET mice were characterized by an overabundance of poorly differentiated myeloblasts with high nuclear/cytoplasm ratios indicative of the impaired differentiation and proliferation associated with the AML-like HSC and MPP phenotypes seen in the BM. Similar to the BM, PRL2-KO;PTEN HET animals did not demonstrate any of these phenotypes and, instead, had leukocytes more similar to WT and PRL2-KO groups. FACS analysis confirmed these observations by identifying an overabundance of myeloid-like populations in the blood samples of PTEN HET animals that was completely ablated in the PRL2-KO;PTEN HET (Figure 4B). Circulating leukocyte populations were further analyzed using FACS to verify that changes were restricted to myeloid lineages. Consistent with observations from the BM, there were no obvious changes seen in circulating lymphoid populations, which would have been reflective of the concurrent development of ALL or other types of leukemia (Supplemental Figure 4B).

### PRL2 deletion reduces splenomegaly as well as AML-associated tissue infiltration.

**Figure 5:**
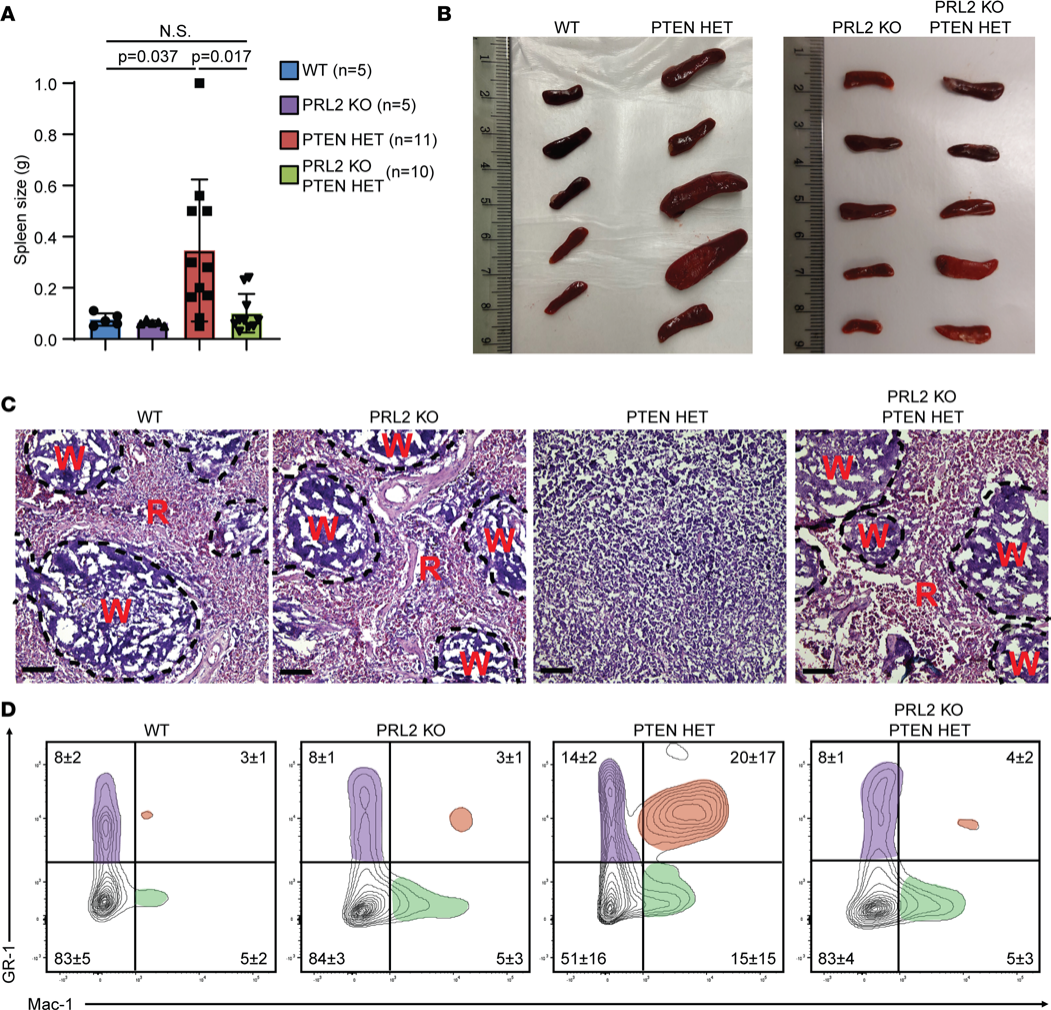
PRL2 deletion significantly reduces splenomegaly development in age-matched mouse groups. (**A**) Quantification of spleen weight between 40-week mice with given *P* values. (**B**) Spleens used to quantify weight. (**C**) Representative microscopy of age-matched spleen samples from each mouse group. Red pulp (R) and white pulp (W) regions are indicated. Visualized with H&E staining. Scale bars: 100 μm. (**D**) Representative FACS analysis of 40-week age-matched splenocytes using myeloid markers. Unique population clusters highlighted with red, green, or purple. Relative abundance of populations quantified for each quadrant based on sample sizes of WT, *n* = 13; PTEN HET, *n* = 5; PRL2 KO, *n* = 6; PRL2-KO;PTEN HET, *n* = 7. Statistical significance was calculated using 1-way ANOVA with a post hoc Tukey’s HSD test.

A common phenotype of advanced AML is the spread of disease from the BM and blood into lymphoid tissues and other organs, such as the spleen and liver, resulting in organ failure and other adverse health events. Animals from all groups were therefore autopsied at death to identify potential tissues suffering from leukemia blast infiltration. Spleens were analyzed from age-matched 40-week postinduction animals to identify leukemia blast infiltration, where no significant change in size was observed between WT (76 mg) and PRL2-KO (63 mg) animals (Figure 5, A and B). By contrast, the PTEN HET animals exhibited considerable splenomegaly (346 mg), while the spleens of PRL2-KO;PTEN HET animals (101 mg) were statistically similar to the WT and PRL2-KO groups and differed significantly from PTEN HET. Samples were analyzed to determine if changes in size were due to changes in cellularity that would be expected of AML due to the proliferation of leukemia blasts. It was determined that splenomegaly in the PTEN HET animals was indeed due to greatly increased splenic cellularity, enough that there was a complete loss of distinction between red pulp and white pulp (Figure 5C). FACS analyses of splenocytes indicate a significant expansion of GR-1^+^Mac-1^+^ monocytic populations in PTEN HET animals, consistent with leukemia blast infiltration resulting in increased splenic cellularity (Figure 5D). As would be expected from the more normal hematopoiesis seen in the BM, PRL2-KO;PTEN HET animals demonstrated none of the phenotypes seen in the PTEN HET animals at this time point. Lymphoid cell populations within the spleen showed no notable disturbances between the different treatment groups, once more showing that this phenotype was restricted to myeloid lineages, as would be expected of AML (Supplemental Figure 4C). Thymus populations were also analyzed to confirm myeloid lineage restriction, and no apparent changes to developing T cell populations were observed (Supplemental Figure 5A). Thymus size was also largely unchanged at 40 weeks after induction, averaging 20–50 mg across each treatment group (our unpublished observations). The liver is also a site of leukemia blast infiltration in late stages of AML disease progression. Histological examination of the liver samples revealed that PRL2 deletion had an attenuating effect on the severity of leukemia blast infiltration (Supplemental Figure 5B). No evidence of infiltration was observed in WT, PRL2-KO, or PRL2-KO;PTEN HET mice, whereas severe leukemia blast infiltration throughout the liver tissue was detected in some PTEN HET mice.The detailed lymphoid analyses indicate that the majority of the PTEN HET animals in this model system are experiencing a severe AML-specific disease phenotype, with a minimally occurring population only possessing ALL characteristics instead. The data derived from the tissue analysis are consistent with the observed changes in HSC populations as presented above and further demonstrate that the effect of elevated PTEN protein level (as in PRL2-KO;PTEN HET animals) in reducing the developing an AML phenotype extended to even more mature myeloid cells beyond of the BM niche.

The detailed lymphoid analyses indicate that the majority of the PTEN HET animals in this model system are experiencing a severe AML-specific disease phenotype, with a minimally occurring population only possessing ALL characteristics instead. The data derived from the tissue analysis are consistent with the observed changes in HSC populations as presented above and further demonstrate that the effect of elevated PTEN protein level (as in PRL2-KO;PTEN HET animals) in reducing the developing an AML phenotype extended to even more mature myeloid cells beyond of the BM niche.

### PRL2 deletion significantly delays the AML phenotype in aged PTEN HET transgenic mice.

**Figure 6:**
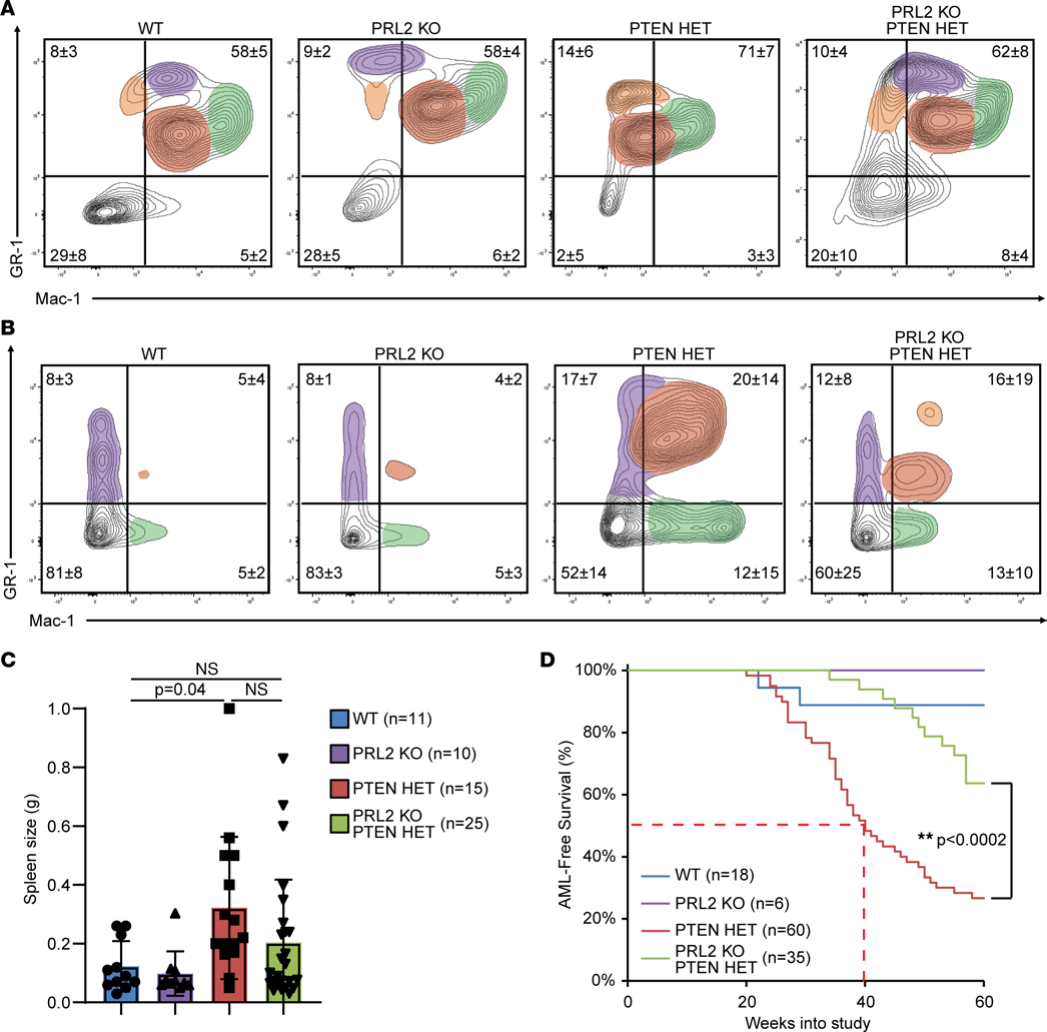
PRL2 deletion significantly delays AML progression in PTEN HET animals and does not fully attenuate disease. (**A**) Representative FACS analysis of residential mature myeloid cell markers in 60-week postinduction bone marrow. Samples size of WT, *n* = 13; PTEN HET, *n* = 9; PRL2 KO, *n* = 5; PRL2-KO;PTEN HET, *n* = 15. (**B**) Representative FACS analysis of 60-week postinduction mouse splenocytes using myeloid markers. WT, *n* = 18; PTEN HET, *n* = 6; PRL2 KO, *n* = 8; PRL2-KO;PTEN HET, *n* = 11. (**C**) Quantification of changes in spleen weight between 60-week postinduction mice with given *P* value. Statistical significance was calculated using 1-way ANOVA with a post hoc Tukey’s HSD test. (**D**) Kaplan-Meier survival plot of assessed AML-free survival in mouse groups after AML induction. Median survival time indicated with dashed line. **P < 0.0002. Statistical significance was calculated using Kaplan-Meier log-rank test.

The 40-week postinduction studies strongly indicate that deletion of PRL2 in the PTEN HET mice imparts a phenotype to the HSC and myeloid populations, within the BM, blood, and spleen, that is more characteristic of WT than that of AML seen in their PTEN HET counterparts. It remains to be addressed, however, the extent to which PRL2 deletion is able to delay AML progression in the PTEN HET animal, as the PTEN protein level was elevated but not fully restored in PRL2-KO;PTEN HET mice (Figure 1C and Figure 2B). Similarly, AKT/mTOR/S6K phosphorylation levels were attenuated but were still higher in the PRL2-KO;PTEN HET mice than in WT. Therefore, mature myeloid populations within the BM of 60-week postinduction animals were characterized similarly to their 40-week counterparts to better determine the long-term protective properties of the PRL2 deletion. WT and PRL2-KO mice continued to exhibit distinct myeloid population clusters, consistent with the populations previously observed in the 40-week postinduction samples (Figure 6A). In contrast, both PTEN HET and PRL2-KO;PTEN HET samples began to show pronounced alterations in population clusters, indicating similarly impaired hematopoiesis. AML development in 60-week postinduction PRL2-KO;PTEN HET animals was further observed within the spleen, where an expansion of leukemoblastic GR-1^+^Mac-1^+^ monocytic populations was observed that was not present in the 40-week samples (Figure 6B). The effect of this population expansion resulted in 60-week PRL2-KO;PTEN HET animals experiencing severe splenomegaly when compared with 60-week WT and PRL2-KO animals (Figure 6C). Compared with WT (122 mg) and PRL2 KO (128 mg), a statistically significant increase in spleen mass was observed in PTEN HET (330 mg), while a large but statistically insignificant increase was observed in PRL2-KO;PTEN HET (230 mg) animal groups. Importantly, and in contrast to the 40-week time point, at 60 weeks after induction, there was no longer a statistically significant difference in spleen size between PTEN HET and PRL2-KO;PTEN HET. Given that the composition and morphology of myeloid populations of the PRL2-KO;PTEN HET animals more closely resembled that of the WT and PRL2-KO animals at 40 weeks — but, in the longer term (>60 weeks), exhibited AML characteristics — the mechanism by which PTEN restoration through PRL2 deletion improves survival appeared not to be by preventing the formation of LSCs but rather by reducing the rate of HSC to LSC transition and the accumulation of LSC-derived leukemia blasts that progressed the AML phenotype.

This conclusion is supported by necropsy data taken from each animal at the time of death. While AML was the most commonly observed cancer in PTEN HET animals, other off-target cancers were observed (Supplemental Table 2). The most common non-AML cancers in PTEN HET and PRL2-KO;PTEN HET mice were ALL and nonmelanoma skin cancer. The development of these cancers was not unexpected, given the use of radiation in the treatment protocol; both ALL and skin cancer arise from mutations in stem cell populations, which, as a result of their high proliferation rate relative to other somatic cells types, are especially sensitive to radiation-induced DNA (45). Regardless, at the PTEN HET median AML-free survival time of 40 weeks, there had been comparatively fewer PRL2-KO;PTEN HET deaths (Figure 6D). By 60 weeks after induction, 88% of PTEN HET animals developed severe AML as well as numerous AML-associated phenotypes. In contrast, and consistent with the extended median survival, the prevalence of AML phenotypes was significantly reduced in WT, PRL2-KO, and PRL2-KO;PTEN HET groups, with only ~40% of the PRL2-KO;PTEN HET mice exhibiting a severe AML phenotype at 60 weeks. Taken together, the survival and phenotypic data demonstrate that PRL2 deletion in the PTEN HET irradiated animals resulted in an increase in PTEN and a concomitant improvement in AML-free survival. In addition, no deleterious effects arising from adult-onset deletion of PRL2 were observed based on the PRL2-KO mice when compared with WT mice.

## Discussion

The PTPs are a diverse and complex family of phosphatases that include the PRLs and PTEN. While the physiological function of PRLs is not currently well understood, they have nonetheless been identified as oncogenes in a variety of cancers (5). PTEN is a known tumor suppressor and regulates numerous cellular processes, including metabolism, motility, proliferation, and survival (25). It was recently demonstrated that PTEN is also a substrate for PRL2 and that dephosphorylation of PTEN Y336 by PRL2 results in increased ubiquitination and proteasomal turnover of the PTEN protein (7). Given the involvement of PTEN in numerous signaling pathways, dysregulation of PTEN by increased protein turnover would be anticipated to have a positive impact on the tumor suppressor activities of PTEN. AML provides an excellent disease model to further investigate the relationship between PRL2 and PTEN in tumorigenesis, since PTEN is frequently observed to be downregulated in the disease, while overexpression of PRL2 is a prognostic marker of adverse clinical outcomes in patients with AML. In vivo demonstration of the effect of PRL2 regulation of PTEN on acute myeloid leukemogenesis would represent an underexplored therapeutic approach in the treatment of the disease.

In the current study, a genetic mouse model was developed to investigate the regulation of PTEN by PRL2 in the suppression of an AML-like leukemic phenotype. Prior studies of the role of PTEN in AML have predominantly been performed in vitro or have utilized PTEN-null mouse models that are incapable of interrogating PTEN activity during AML onset and progression. The conditional PTEN heterozygous, radiation-induced AML model generated in the current study enabled the examination of the effect of subtle changes in PTEN protein level on HSC pathogenesis and represents the first report to our knowledge of such a conditional AML model. The PTEN HET mice exhibited an overall AML phenotype comparable with previously established transgenic AML models: stem cell morphology reflected a reduction in normal HSCs, there was a general shift in MPP cell populations toward GMP lineages, there was an accumulation of clinically relevant (>20%) concentrations of blast-like cells within the BM, and there was infiltration of these blast-like cells to distal tissues such as the spleen (46). AML development and mortality occurred within a physiologically relevant time frame and was similar to previously published single-mutation transgenic AML models (34, 35). Furthermore, the model, based on spontaneous, radiation-induced AML generating mutations (45), should more accurately reflect the complex cytogenic phenotypes observed in patients with AML, rather than single- or double-hit mutations as used in other models. This provides for more accurate analysis of the role of PTEN in suppressing the AML disease progression. Nonetheless, while the irradiated PTEN HET model produces a phenotype with many characteristic hallmarks of AML, it has yet to be conclusively demonstrated that the blast cells generated are fully transformed AML blasts and not some form of myeloproliferative neoplasms.

PRL2 has been shown to negatively regulate PTEN protein concentration, and constitutive PRL2 KO in a PTEN-deficient animal model demonstrated enhanced tumor-free survival coincident with elevated PTEN protein levels compared with PRL2 WT animals (7). However, the phenotype observed in that constitutive model may be confounded by compensatory mechanisms arising from PRL2 deletion and PTEN deficiency during development. Thus, it remained to be determined whether an acute or chronic PRL2 loss would affect tumorigenesis. The generation of the conditional PRL2-floxed mouse model allowed for normal PRL2 expression throughout development while enabling temporal transgene expression relevant to age-dependent diseases such as AML. The characteristics of the model, therefore, provide an improved and broad platform for validating the physiological effect of PRL2 inhibition on PTEN and oncogenesis. In the current study of AML in PTEN-deficient mice, PRL2 deletion delayed the disease progression and severity. Comprehensive analysis of the myeloid cell populations, after AML induction, revealed that the expansion of poorly differentiated myeloid stem cells in the BM was significantly reduced in the PRL2-KO cohort and was coincident with a relative 20% increase in PTEN protein abundance in these animals. Consistent with the relative increase in the amount of PTEN, biochemical analyses further indicated suppression of the AKT pathway and reduced cell proliferation. These observations provide further evidence of the role of PRL2 in modulating PTEN protein turnover and, as a consequence, the activity of PTEN-regulated pathways. Furthermore, the study confirmed that the conditional, temporal loss of PRL2 was capable of delaying the disease pathogenesis, and it supports PTEN augmentation by PRL2 inhibition as a potential therapeutic strategy. However advancement of the approach toward clinical application will require further investigation into the fitness and repopulation potential of HSCs following PRL2 inhibition over the course of disease pathogenesis.

It is well recognized that abnormal activation of the PI3K/AKT/mTOR pathway, either directly or indirectly, is critical to the onset and development of AML (38). However, the complexity of upstream activators and downstream effectors of AKT, together with the feedback loops and crosstalk between multiple pathways within the BM niche, has hindered the development and clinical application of therapies that provide long-term efficacy (37, 38). Simultaneously targeting multiple pathways using combinatorial therapy has yet to yield a viable approach, and the ability to overcome the development of drug resistance remains a considerable obstacle to successful therapeutic intervention. In addition to the pathway and network complexity, the development of specific pharmacological inhibitors of key enzymes in the PI3K/AKT/mTOR pathway is hampered by suboptimal therapeutic efficacy and both on- and off-target toxicities (47). PTEN is a well-established antagonist of the PI3K/AKT pathway; by dephosphorylating PIP3 to PIP2, PTEN prevents the activation of AKT and impairs AKT downstream signaling. As a result, reduction in PTEN leads to a concomitant progressive dose-dependent activation of the PI3K/AKT pathway. It has been long known that an inverse correlation exists between PTEN abundance and AKT pathway activity in patients with hematological malignancies (48), and reducing AKT signaling in LSCs through upregulating PTEN protein level has been previously suggested as a potential therapeutic approach for treating AML (37). Elevation of PTEN protein levels by PRL2 inhibition has been shown in the current study to prolong the time necessary for development of leukemogenesis and the progression of an AML phenotype by downregulating the AKT/mTOR signaling. PRL2 inhibition, therefore, provides a mechanism and potential therapeutic strategy to elevate the level of PTEN protein, attenuate hyperactivated AKT signaling, and rescue the AML phenotype. It is also notable that the reduction in PI3K/AKT/mTOR signaling was evident in all PRL2-KO;PTEN HET samples analyzed. Given the random nature of the radiation-induced mutations in the PTEN HET model system driving AKT activation, PTEN augmentation via PRL2 inhibition appears to have broad therapeutic efficacy. This potential efficacy was recently demonstrated using an FLT3-ITD AML model; these models are known to possess constitutively active AKT signaling. FLT3 mutations are one of the more common AML-associated lesions, and using ectopic FLT3-ITD cell lines, in both in vitro and in vivo transplantation studies, it was demonstrated that PRL2 inhibition reduced AML burden in these FLT3-driven AMLs (49).

The mutational complexity and resulting oncogenic signaling in patients with AML have hampered the clinical translation of many promising in vitro and preclinical drug discovery leads. The current study explored what is believed to be a novel AML therapeutic approach and validated that oncogenic signaling during AML disease development could be attenuated by PTEN protein augmentation via PRL2 depletion. Similar to AML, a number of cancers exhibit increased AKT pathway activity coincident with reduced levels of PTEN protein. While targeting the PI3K/AKT pathway has been a common therapeutic approach in these PTEN-deficient cancers, it is often limited by the severe on- and off-target effects arising from crosstalk between AKT-regulated pathways (50). Therapeutic restoration of PTEN protein via inhibition of PRL2 would therefore represent a direct strategy for attenuating aberrant PI3K/AKT signaling. PTEN regulated processes, such as DNA damage repair and cell cycle regulation, may also benefit from the reestablishment of PTEN protein levels. Furthermore, comparison of the WT and PRL2-KO animals in the current study revealed no consequential deleterious effects of PRL2 deletion. Indeed, the approximate 20% increase in PTEN protein observed was beneficial to long-term animal survival. The apparent lack of consequential adverse side effects suggests that PRL2-specific inhibition may be advantageous to the development of combinatorial therapies to address a broad range of PTEN-deficient cancers. While the results are promising, future investigation of the potential of PRL2 inhibition as a therapeutic strategy for AML and other cancers will require investigation and validation of the efficacy of the approach during both early-stage development and progression, as demonstrated in the current study, and also the impact of PRL2 inhibition of late-stage disease. Furthermore, given that PRL2 inhibition was achieved genetically in a complete KO model, any comparable small-molecule inhibitor must, by necessity, substantially inhibit PRL2 activity, as previous in vivo and in vitro studies interrogating PRL2 heterozygous deletions demonstrated no discernible phenotypic difference or significant changes in the quantity of PTEN protein from PRL2 WT (37). Furthermore, Chen et al. recently identified CBL, an E3 ligase, as a direct substrate of PRL2 (49). This reflected a mechanism of action whereby PRL2 dephosphorylation of CBL Y371 inhibited CBL-E3 ligase activity, resulting in decreased FLT3 ubiquitination and protein degradation to sustain FLT3-ITD driven oncogenic signaling. These findings together with the current study demonstrate that PRL2 regulates turnover of at least 2 key targets in AML, the PTEN tumor suppressor and the oncogenic FLT3 receptor. Notably, it was also observed that AML samples containing WT FLT3 were also sensitive to PRL2 inhibition, a result that would be consistent with the additional activity of PRL2 on PTEN such as demonstrated in the current study. The combination of results from the 2 studies provides compelling evidence for the continued development of novel PRL2 inhibitors.

## Methods

### Reagents and antibodies.

Tamoxifen (CAS 10540-29-1) was obtained from as a powder from Cayman Chemicals (catalog 13258). A TUNEL Apo-Green Detection Kit from Biotool was used (Biotool, catalog B31115). The following antibodies used for FACS analysis were obtained from BioLegend: B220-488 (catalog 103225), CD3-647 (catalog 100209), CD4-PeCy5 (catalog 100514), CD8-BV711 (catalog 100748), CD11b-BV711 (catalog 101242), CD11c-647 (catalog 117312), CD16/32 (catalog 101302), CD16/32-PE (catalog 156606), CD19-PE (catalog 115508), CD34-647 (catalog 152205), CD45-PeCy7 (catalog 103114), CD117-BV421 (catalog 105828), Ki67-PE (catalog 151210), Ly6C-PE (catalog 128007), Ly6G-488 (catalog 127626), Sca1-BV711 (catalog 108131), and Lin cocktail (catalog 133302). Primary antibodies used for Western blotting were: PRL1/2 (provided by Qi Zheng, A-STAR Institute of Molecular Cell Biology, Singapore, generated as previously described; ref. 36), PTEN (Cell Signaling Technology catalog 9188, RRID:AB_2253290), PTEN Y336 (NSJ Bioreagents, catalog F48744), AKT (Cell Signaling Technology, catalog 9272, RRID:AB_329827), AKT S473 (Cell Signaling Technology, catalog 4060, RRID:AB_2315049), MTOR (Cell Signaling Technology, catalog 2972, RRID:AB_330978), MTOR S2448 (Cell Signaling Technology, catalog 2971, RRID:AB_330970), S6K (Cell Signaling Technology, catalog 9202, RRID:AB_331676), S6K T389 (Cell Signaling Technology, catalog 9205, RRID:AB_330944), β-actin (Santa Cruz Biotechnology, catalog sc-47778, RRID:AB_626632), PARP(Cell Signaling Technology, catalog 9542, RRID:AB_2160739), Caspase 3 (Cell Signaling Technology, catalog 14220, RRID:AB_2798429), Cleaved Caspase 3 (Cell Signaling Technology, catalog 9661, RRID:AB_2341188), Caspase 9 (Cell Signaling Technology, catalog 9508, RRID:AB_2068620), MDM2 (Cell Signaling Technology, catalog 86934, RRID:AB_2784534), MDM2 S166 (Cell Signaling Technology, catalog 3521, RRID:AB_2143550), and BAD S136 (Cell Signaling Technology, catalog 4366, RRID:AB_10547878). Secondary antibodies used were: anti–rabbit IgG, HRP-linked (Cell Signaling Technology, catalog 7074, RRID:AB_2099233), and anti–mouse IgG, HRP-linked (Cell Signaling Technology, catalog 7076, RRID:AB_330924).

### Transgenic mice.

This study utilized commercially available C57BL/6J Rosa26-CreER^T2^ (catalog 008463, RRID:IMSR_JAX:008463) and 129S4/SvJae PTEN^fl/fl^(catalog 004597, RRID:IMSR_JAX:004597) mice from The Jackson Laboratory. A *Prl2*^fl/fl^ mouse was generated within WT C57BL/6J strain mice from The Jackson Laboratory in house using CRISPR/Cas9 recombination as described. Animals were backcrossed for 3 generations onto a C57BL/6J background prior to inclusion in study. Equal numbers of male and female mice were enrolled in each study; unless otherwise noted, no statistical difference between sexes was observed.

### Genotyping.

DNA was isolated from 1 mm tail snips taken from mouse pups during weaning. Tails snips were boiled at 95°C for 20 minutes in a solution of 25 mM NaOH and 200 μM EDTA buffered to pH 12. The solution was cooled to room temperature and then neutralized by the addition of equal volume 40 mM Tris-HCl, pH 5. The following primer sets were used to identify transgenes: Rosa26 WT forward: 5′-CTG GCT TCT GAG GAC CG-3′, reverse: 5′-CCG AAA ATC TGT GGG AAG TC-3′; Rosa26-CreER^T2^ forward: 5′-CGT GAT CTG CAA CTC CAG TC-3′, reverse: 5′-AGG CAA ATT TTG GTG TAC GG-3′; *Pten*^fl/fl^ forward: 5′-CAA GCA CTC TGC GAA CTG AG-3′, reverse: 5′-AAG TTT TTG AAG GCA AGA TGC-3′; *Prl2*^fl/fl^ forward: 5′-CAC ACA CTT AAG TAA GTA CCT GGT TGG-3′, reverse: 5′-CCA ATC ATC TAC TAT CTG ATT AGG G-3′. PCR reaction mixtures were made using GoTaq Flexi DNA Polymerase (Promega, catalog M8295), and cycling protocols were performed according to The Jackson Laboratory recommendations. The in-house *Prl2*^fl/fl^ cycling conditions were as follows: 95°C 5 minutes, (95°C 30 seconds, 57°C 45 seconds, 72°C 1 minute) ***×*** 34, 72°C 5 minutes, 4°C hold.

### Sample preparation for Western blot analyses.

Tissue samples were homogenized on dry ice using a lysis buffer made of 50 mM Tris-HCl, 150 mM NaCl, 10% glycerol, and 0.5% Triton X-100 containing a cocktail of protease inhibitors (Roche, catalog 04693132001). Protein concentration was quantified by Bradford assay using the Coomassie Plus Protein Assay kit (Thermo Fisher Scientific, catalog 23236). Samples were electrophoresed on 7.5%–15% SDS-PAGE gels using the Bio-Rad Mini-PROTEAN gel casting system. Samples were transferred to nitrocellulose membranes, blocked with 5% w/v BSA in TBST, and then incubated with primary antibodies at a 1:1,000–1:2,000 dilution in TBST overnight at 4°C. Membranes were washed and then incubated with HRP-conjugated secondary antibodies at a 1:3,000–1:5,000 dilution in TBST overnight at 4°C. Membranes were washed and then visualized using SuperSignal West Pico (Thermo Fisher Scientific, catalog 34580), and SuperSignal West Femto (Thermo Fisher Scientific, catalog 34095) Chemiluminescent Substrate as necessary.

### Tamoxifen treatment.

Tamoxifen powder was dissolved in corn oil (Sigma-Aldrich, catalog C8267) at a stock concentration of 20 mg/mL by rocking overnight in the dark at 37°C. This stock solution was then filtered through a 70 mm mesh filter (Falcon, catalog 352350) and stored at 4°C until necessary. Animals were given i.p. injections of tamoxifen at the concentration indicated for each protocol using sterile 25 G needles (BD Biosciences, catalog 305125), alternating the injection site between the right and left sides of the thoracic cavity to minimize local tissue damage.

### Radiation induction.

Animals from 10–20 weeks of age were individually exposed to a full-body dose of radiation originating from cobalt-60 γ radiation source for the given experimental dosage in Grays. All safety precautions and guidelines were followed as required by Purdue Radiation and Environmental Management.

### Analyses of leukemia disease progression and severity.

Following AML induction, animals are inspected daily and weighed weekly in order to allow us to identify changes in weight or overall health caused by leukemia-induced discomfort. A loss of 20% body weight is the maximum amount allowed by the Purdue IACUC and was used as the experimental endpoint for the mouse model. Upon reaching 20% weight loss from the start of the study, animals were euthanized via asphyxiation using CO_2_ or anesthetic overdose using isoflurane. The following tissue samples were then collected: whole blood via cardiac puncture, thymus, thyroid, lymph nodes (inguinal, deep cervical, superficial cervical, and brachial), spleen, both femurs, 2 lobes from the liver, both kidneys, both adrenal glands, and any other specific tissue that appeared abnormal or cancerous. Tissue not immediately used for protein or cellular analysis was snap frozen in liquid nitrogen and stored at –80°C or fixed in 4% PFA and then stored at 4°C. Animal samples were segregated for analysis based on whether animal death had resulted primarily from AML or non-AML drivers, with the latter being excluded from phenotypic and statistical consideration.

### BM isolation.

To isolate BM for analysis, the left and right femurs were removed from mice and cleaned of attached muscle tissue and cartilage during necropsy. The femur is then cut above the kneecap and immediately below the junction with the hip to expose the medullary cavity at both ends. The femurs were added to a 500 μL tube with a narrow hole pierced into the bottom, and the tube was then placed into a 1.5 mL microfuge tube. The nested tubes were briefly spun down for 10 seconds at 3,500*g* with their lids open. The empty femurs remained in the 500 μL tube while the BM collected in the bottom of the 1.5 mL tube. BM isolated this way could then be resuspended in lysis buffer for protein analysis, resuspended in 3% BSA + 5 mM EDTA for FACS analysis, or applied onto a microscope slide (Thermo Fisher Scientific, catalog 12-550-15) using a natural bristle paintbrush for microscopy. BM microscopy slides were fixed with 6 dips into 100% methanol for long-term storage.

### Peripheral blood smears.

Whole blood collected from a cardiac puncture was added to a tube containing trace amounts of 5 mM EDTA to inhibit blood clotting. Wedge smears of the blood were prepared by adding 1–3 μL of whole blood to the top of a charged microscope slide (Thermo Fisher Scientific, catalog 12-550-15), the back of a “spreader” slide was angled into the drop so it spread evenly across the width of the slide, and the drop was then “pulled” to along the length using the spreader slide. This created a thin film with a noticeable feathered edge possessing a rainbow sheen. Peripheral blood smears were then fixed with 6 dips into 100% methanol for long-term storage.

### Tissue sectioning.

Fixed spleen and liver tissue was mounted in OCT compound (Thermo Fisher Scientific, catalog 4585) and serially sectioned at 7 mm thickness onto charged microscope slides using a Leica CM1850 cryostat. Slides were then left at –20°C for long-term storage.

### Tissue staining and microscopy.

Blood and BM samples were stained for microscopy by immersing the methanol-fixed slides into a 1:20 dilution of Wright-Giesma stain (Sigma-Aldrich, catalog 32884) in PBS for 20 minutes and then destaining in distilled water to preference. Liver and spleen tissue was instead stained with Mayer’s Hematoxylin (Sigma-Aldrich, catalog MHS16) for 5 minutes, counter-stained with Eosin Y (Sigma-Aldrich, catalog 588X) for 1 minute, and then destained in distilled water. Slides were imaged using a Leica DM6B microscope with DFC450 color camera. Microscopy images were then processed using Leica Application Suite X software. Severity of BM blast percentage was assessed via microscopy using IPSS guidelines (good, < 5%; intermediate, 5%–10%; poor, 11%–30%). Morphology of leukemia blasts was determined using guidelines and reference materials from MedSci, Corpath, PathologyOutlines, and assorted publication guides (51).

### Sample preparation for FACS analysis.

To perform FACS analysis, spleen and thymus samples were first reduced to single-cell suspensions by compressing the tissue through a 70 mm filter (Falcon, catalog 352350) into cold 1***×*** PBS supplemented with 5 mM EDTA using the plunger of a syringe (HSW, catalog 4010-200V0). Samples were strained through a second filter and then resuspended in cold blocking buffer made of 5% BSA, 0.5 mM EDTA, and a 1:1,000 dilution of CD16/32. Whole blood collected via cardiac puncture was prepared by lysing RBCs in a buffer made with 155 mM NH_4_Cl, 12 mM NaHCO_3_, and 0.1 mM EDTA for 1 minute; washing in 1***×*** PBS; and then resuspending in blocking buffer. BM cells could be immediately resuspended in blocking buffer after acquisition. Samples were then fixed in 4% PFA, depending on the antibodies being used, or immediately began incubation with fluorophore-conjugated antibodies. Samples were incubated with antibodies at 1:600 dilution in blocking buffer on ice for 2 hours. The following antibody combinations were used for population analysis. BM HSCs: Lin cocktail, c-Kit, Sca-1, CD16/32, CD34, and CD45; adaptive immune cells: CD45, CD3, CD4, CD19, and B220; innate immune cells: CD45, CD11b, CD11c, Ly6C, and Ly6G; proliferation: CD45, CD11b, CD11c, Ki67, and Ly6G; and apoptosis: CD45, CD11b, CD11c, Ly6C, and TUNEL. Afterward, staining samples were washed and resuspended in 1***×*** PBS before being added to 5 mL polystyrene tube for analysis (Falcon, catalog 352235). Sample data were acquired using a BD LSRFortessa Cell Analyzer, and results were analyzed using FlowJo. Proliferation was determined by quantifying the number of Ki67^+^ cells staining for myeloid lineage markers, while apoptosis was determined by quantifying the number of TUNEL^+^ cells within these same populations.

### Statistics.

Western blot data were quantified for statistical analysis using ImageJ software. Protein abundance was determined as black pixel intensity after subtracting background. Protein concentration was determined relative to loading control concentration. Phosphorylated protein concentration was determined by concentration relative to total protein. Significant differences were determined in protein concentration data sets and spleen weights using 1-way ANOVA with post hoc Tukey’s honestly significant difference (HSD) correction for multiple comparisons. Survival analysis was performed using the Kaplan-Meier log-rank test. Statistics for phenotype at endpoint were determined using a χ^2^ comparison of proportions. Data are shown as mean ± SD for the given number of replicates. *P* < 0.05 was considered statistically significant.

### Study approval.

All animal experiments were conducted in compliance with Purdue IACUC approved protocols (protocol no. 1511001324). Animals were housed and cared for in accordance with Purdue IACUC guidelines.

### Data availability.

The data values used to generate plots for this study are in the Supporting Data Values file; files for raw data and microscopy images are available upon request from the corresponding author.

## Author contributions

CC and ZYZ designed the research study, with input from YB, APH, and FNM. CC and APH conducted the primary experiments, acquired data, and performed data analysis. YB and FNM conducted supplemental experiments and provided data. YB and QL provided reagents. QL was responsible for overseeing generation of transgenic PRL2-floxed allele. ZYZ provided financial support and overall guidance. CC and ZYZ wrote the manuscript, with input from YB.

## Supplementary Material

Supplemental data

Supporting data values

## Version 1 09/04/2023

In-Press Preview

## Version 2 10/09/2023

Electronic publication

## References

[1] Yu ZH, Zhang ZY (2018). Regulatory mechanisms and novel therapeutic targeting strategies for protein tyrosine phosphatases. Chem Rev.

[2] Bollu LR (2017). Molecular pathways: targeting protein tyrosine phosphatases in cancer. Clin Cancer Res.

[3] Stephens BJ (2005). PRL phosphatases as potential molecular targets in cancer. Mol Cancer Ther.

[4] Campbell AM, Zhang Z-Y (2014). Phosphatase of regenerating liver: a novel target for cancer therapy. Expert Opin Ther Targets.

[5] Hardy S (2018). Physiological and oncogenic roles of the PRL phosphatases. FEBS J.

[6] Kobayashi M (2014). Phosphatase of regenerating liver in hematopoietic stem cells and hematological malignancies. Cell Cycle.

[7] Li Q (2020). Mechanism of PRL2 phosphatase-mediated PTEN degradation and tumorigenesis. Proc Natl Acad Sci U S A.

[8] Hollander MC (2011). PTEN loss in the continuum of common cancers, rare syndromes and mouse models. Nat Rev Cancer.

[9] Fitzmaurice C (2019). Global, regional, and national cancer incidence, mortality, years of life lost, years lived with disability, and disability-adjusted life-years for 29 cancer groups, 1990 to 2017: a systematic analysis for the Global Burden of Disease Study. JAMA Oncol.

[10] GBD 2019 Diseases (2020). Global burden of 369 diseases and injuries in 204 countries and territories, 1990-2019: a systematic analysis for the Global Burden of Disease Study 2019. Lancet.

[11] Riether C (2015). Regulation of hematopoietic and leukemic stem cells by the immune system. Cell Death Differ.

[12] Grove CS, Vassiliou GS (2014). Acute myeloid leukaemia: a paradigm for the clonal evolution of cancer?. Dis Model Mech.

[13] Mrózek K (2008). Cytogenetic, molecular genetic, and clinical characteristics of acute myeloid leukemia with a complex karyotype. Semin Oncol.

[14] Song H (2019). The expression of PTEN and INPP4B and their clinical significance in patients with acute myeloid leukemia. Eur J Inflamm.

[15] Huang X (2015). Prognostic value of the expression of phosphatase and tensin homolog and CD44 in elderly patients with refractory acute myeloid leukemia. Oncol Lett.

[16] Zhang J (2006). PTEN maintains haematopoietic stem cells and acts in lineage choice and leukaemia prevention. Nature.

[17] Yilmaz ÖH (2006). Pten dependence distinguishes haematopoietic stem cells from leukaemia-initiating cells. Nature.

[18] Cheong J-W (2003). Phosphatase and tensin homologue phosphorylation in the C-terminal regulatory domain is frequently observed in acute myeloid leukaemia and associated with poor clinical outcome. Br J Haematol.

[19] Noguera NI (2013). Nucleophosmin/B26 regulates PTEN through interaction with HAUSP in acute myeloid leukemia. Leukemia.

[20] Bessette DC (2008). PRL PTPs: mediators and markers of cancer progression. Cancer Metastasis Rev.

[21] Bagger FO (2019). BloodSpot: a database of healthy and malignant haematopoiesis updated with purified and single cell mRNA sequencing profiles. Nucleic Acids Res.

[22] Arora D (2012). Expression of protein-tyrosine phosphatases in Acute Myeloid Leukemia cells: FLT3 ITD sustains high levels of DUSP6 expression. Cell Commun Signal.

[23] Kobayashi M (2017). Phosphatase PRL2 promotes AML1-ETO-induced acute myeloid leukemia. Leukemia.

[24] Tate JG (2019). COSMIC: the catalogue of somatic mutations in cancer. Nucleic Acids Res.

[25] Lee Y-R (2018). The functions and regulation of the PTEN tumour suppressor: new modes and prospects. Nat Rev Mol Cell Biol.

[26] Yu H (2010). Relevant mouse model for human monocytic leukemia through Cre/lox-controlled myeloid-specific deletion of PTEN. Leukemia.

[27] Aigner P (2019). STAT3β is a tumor suppressor in acute myeloid leukemia. Blood Adv.

[28] McCormack E (2008). Review: genetic models of acute myeloid leukaemia. Oncogene.

[29] Almosailleakh M, Schwaller J (2019). Murine models of acute myeloid leukaemia. Int J Mol Sci.

[30] Skayneh H (2019). A critical review of animal models used in acute myeloid leukemia pathophysiology. Genes (Basel).

[31] Alimonti A (2010). Subtle variations in Pten dose determine cancer susceptibility. Nat Genet.

[32] Martelli AM (2019). The key roles of PTEN in T-cell acute lymphoblastic leukemia development, progression, and therapeutic response. Cancers (Basel).

[33] Stouten S (2021). Modeling low-dose radiation-induced acute myeloid leukemia in male CBA/H mice. Radiat Environ Biophys.

[34] Schessl C (2005). The AML1-ETO fusion gene and the FLT3 length mutation collaborate in inducing acute leukemia in mice. J Clin Invest.

[35] Rau R (2014). NPMc+ cooperates with Flt3/ITD mutations to cause acute leukemia recapitulating human disease. Exp Hematol.

[36] Dong Y (2014). Phosphatase of regenerating liver 2 (PRL2) deficiency impairs Kit signaling and spermatogenesis. J Biol Chem.

[37] Gurska LM (2019). Signaling pathways in leukemic stem cells. Adv Exp Med Biol.

[38] Nepstad I (2020). The PI3K-Akt-mTOR signaling pathway in human acute myeloid leukemia (AML) cells. Int J Mol Sci.

[39] Ghosh J (2016). S6K1 regulates hematopoietic stem cell self-renewal and leukemia maintenance. J Clin Invest.

[40] Renneville A (2008). Cooperating gene mutations in acute myeloid leukemia: a review of the literature. Leukemia.

[41] Challen GA (2009). Mouse hematopoietic stem cell identification and analysis. Cytometry A.

[42] Agarwal A (2019). Differentiation of leukemic blasts is not completely blocked in acute myeloid leukemia. Proc Natl Acad Sci U S A.

[43] Juntilla MM (2010). AKT1 and AKT2 maintain hematopoietic stem cell function by regulating reactive oxygen species. Blood.

[44] Ngo S (2021). Acute myeloid leukemia maturation lineage influences residual disease and relapse following differentiation therapy. Nat Commun.

[45] Rivina L (2014). Radiation-induced myeloid leukemia in murine models. Hum Genomics.

[46] Ye M (2015). Hematopoietic differentiation is required for initiation of acute myeloid leukemia. Cell Stem Cell.

[47] Janku F (2017). Phosphoinositide 3-kinase (PI3K) pathway inhibitors in solid tumors: From laboratory to patients. Cancer Treat Rev.

[48] Dahia PL (1999). PTEN is inversely correlated with the cell survival factor Akt/PKB and is inactivated via multiple mechanismsin haematological malignancies. Hum Mol Genet.

[49] Chen H (2023). PRL2 phosphatase enhances oncogenic FLT3 signaling via dephosphorylation of the E3 ubiquitin ligase CBL at tyrosine 371. Blood.

[50] Shariati M, Meric-Bernstam F (2019). Targeting AKT for cancer therapy. Expert Opin Investig Drugs.

[51] Zini G (2021). How I investigate difficult cells at the optical microscope. Int J Lab Hematol.

