# Supplementary material for: PRL2 inhibition elevates PTEN protein and ameliorates progression of acute myeloid leukemia
Source: JCI Insight. 2023 Oct 9;8(19):e170065. doi: 10.1172/jci.insight.170065 (PMC10619439; doi:10.1172/jci.insight.170065)
Supplement: Supplemental data [file jciinsight-8-170065-s139.pdf]

PRL2 Inhibition Elevates PTEN Protein and Ameliorates Progression of Acute Myeloid Leukemia

Colin Carlock[1], Yunpeng Bai[2], Allison Paige-Hood[3], Qinglin Li[2#], Frederick Nguele Meke[2], and Zhong-Yin Zhang[2,4,5,6*]

[1]Department of Biochemistry, [2]Department of Medicinal Chemistry and Molecular Pharmacology, [3]Department of Biological Sciences, [4]Department of Chemistry, [5]Institute for Cancer Research, and [6]Institute for Drug Discovery, Purdue University, West Lafayette, IN 47907, USA

[#]Current address: ThermoFisher Scientific, 5781 Van Allen Way, Carlsbad, CA 92008, USA

To whom correspondence should be addressed: Dr. Zhong-Yin Zhang, 720 Clinic Drive, West Lafayette, IN 47907, (765)-496-3720, zhang-zy@purdue.edu

Supplemental Figure 1. Generation of conditional PRL2 knockout transgene. (a) Location of loxP insertion sites within *prl2* gene. (b) Genotypic validation of loxP site insertion at 3' and 5' of exon 4. (c) Breeding scheme to generate floxed allele animal. (d) Genotypic validation of floxed allele in mice. (e) Change in tissue protein concentration of PRL2 and PTEN following Cre-recombination in animals with ROSA26-CreER^{T2} promoter

Supplemental Figure 2. Optimization and analysis of treatment conditions for PTEN HET irradiated mouse model. (a) Different protocols tested for optimal leukemia induction (Gy = Gray, absorbed dose of radiation). (b) Average change in weight from the study's start for PTEN HET in each protocol treatment group compared against the averaged WT weight change from those groups. Similar downward trajectories of weight loss are observed after 15-20 weeks in most treatment groups. (c) Kaplan-Meier plot showing overall survival of PTEN HET treatments group along with combined results of WT mice from each group.

Supplemental Figure 3. No significant changes in apoptosis signaling was observed in the myeloid cells of pre-irradiation animals. (a) Western blot analysis of apoptosis pathway in 1 week post-induction spleen samples from transgenic mouse groups. (b) Quantification of apoptosis pathway protein levels from panel (a), error bars represent three independent experiments. (c) Quantification of apoptosis seen in CD11b+, CD11c+ and GR-1+ myeloid populations of 1 week spleens. (d) Western blot analysis of PTEN Y336 in 1 week post-induction spleen samples from transgenic mouse groups. (e) Quantification of PTEN Y336 changes from panel d. Sample size of n = 3 for each animal group. ** $p<0.02$, *** $p<0.001$, N.S.- no statistical significance. Statistical significance calculated using one-way ANOVA with a post hoc Tukey's HSD test.

Supplemental Figure 4. No significant changes were observed within lymphocyte populations regardless of tissue. (a) Representative FACS of mature lymphocytes obtained from the bone marrow of 40 wk post-induction animals (b) Representative FACS of mature lymphocytes in circulating blood of 40 wk post-induction animals. (c) Representative FACS of mature lymphocytes from spleens of 40 wk post-induction animals. Unique population clusters highlighted with red (CD3+CD8+ T-killer cells), green (CD3+CD4+ T-helper cells), orange (CD19+B220- B cells) or purple (CD19+B220+ plasma cells).

Supplemental Figure 5. No significant changes are seen within the lymphoid cells within the thymus, despite leukemia blast burden becoming high enough to damage the liver. (a) Representative FACS of developing T cells from the thymus of 40 wk post-induction animals from each mouse group. (b) Representative microscopy of liver tissue obtained from 40 wk post-induction mice showing disruption to normal liver structure in PTEN HET mice due to infiltrating leukemia blasts (dark purple cells). Visualized using H&E staining, imaged at 100x magnification.

Supplemental Table 1. Incidence rate of cancer phenotypes at time of death in each mouse group at 36 weeks into the study. Statistical significant calculated relative to WT. Sample size for each group: WT n = 23, PTEN HET n = 41, PRL2 KO n = 7, PRL2 KO;PTEN HET n = 28. *p<0.05, **p<0.01, ***p<0.0001. Statistical significance calculated using chi-square comparison of means.

Supplemental Table 2. Incidence rate of cancer phenotypes at time of death in each mouse group at 60 weeks into the study. A statistical significant calculated relative to WT. Sample size for each group: WT n = 23, PTEN HET n = 59, PRL2 KO n = 9, PRL2 KO;PTEN HET n = 33 *p<0.05, **p<0.01, ***p<0.0001. Statistical significance calculated using chi-square comparison of means.

Supplemental Figure 1

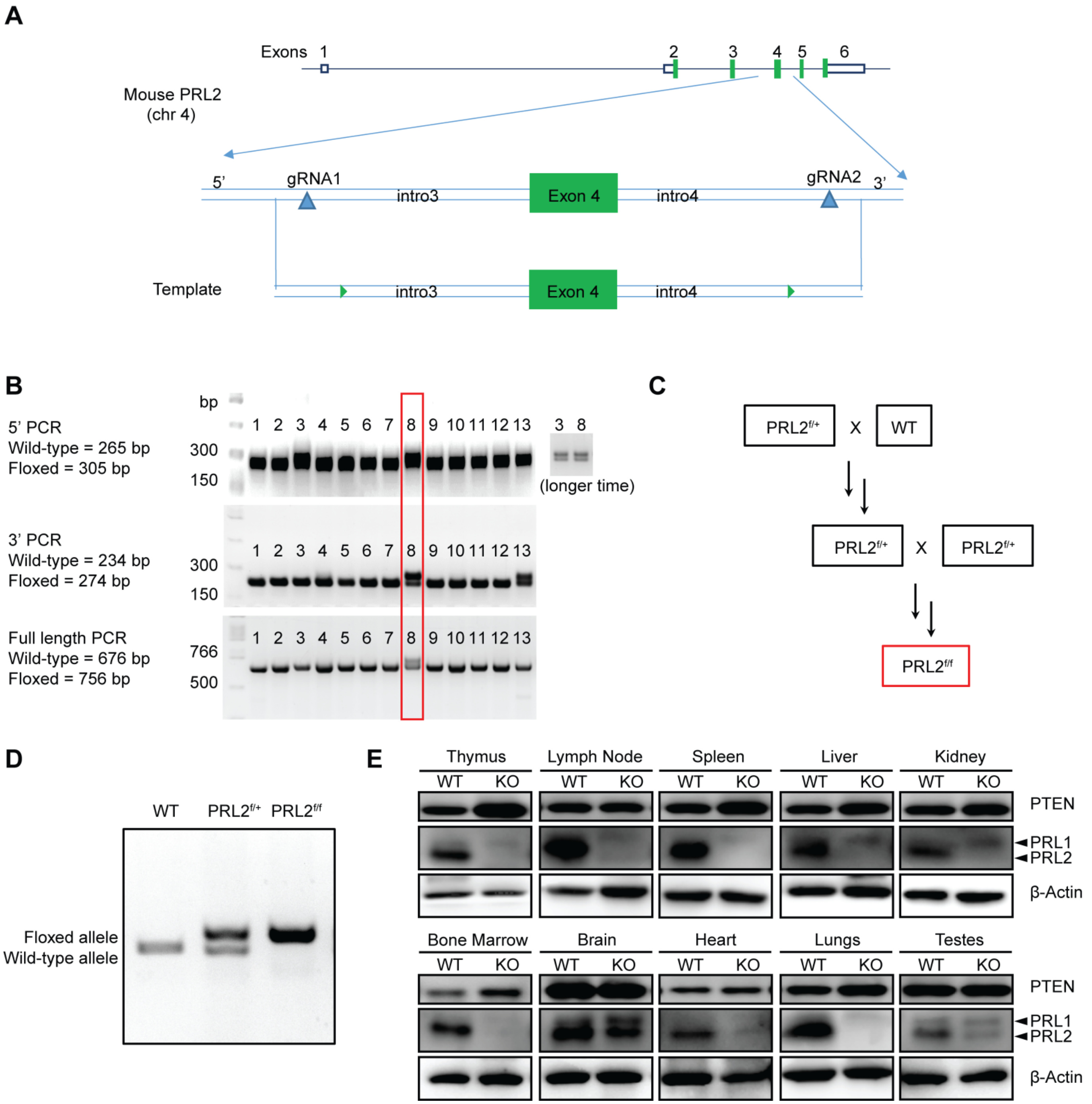

Supplemental Figure 2

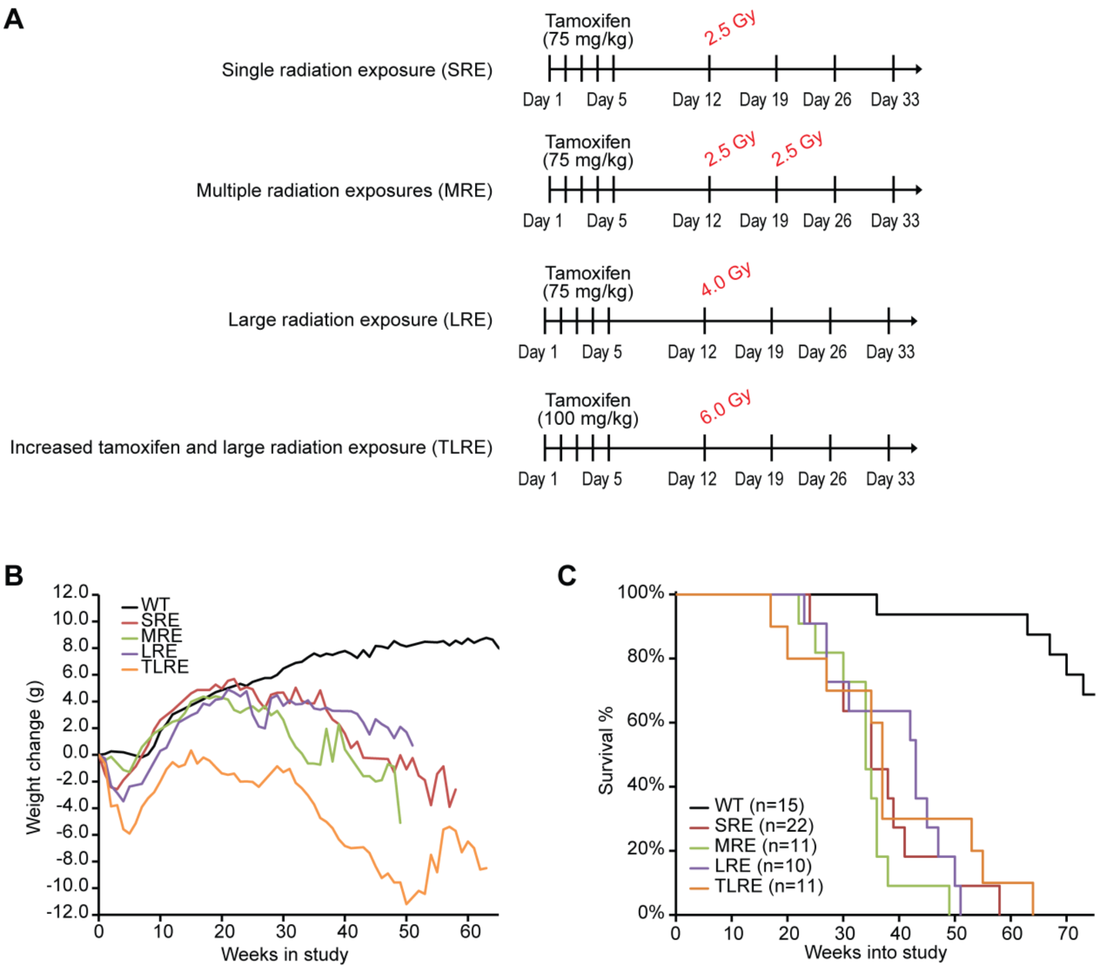

Supplemental Figure 3

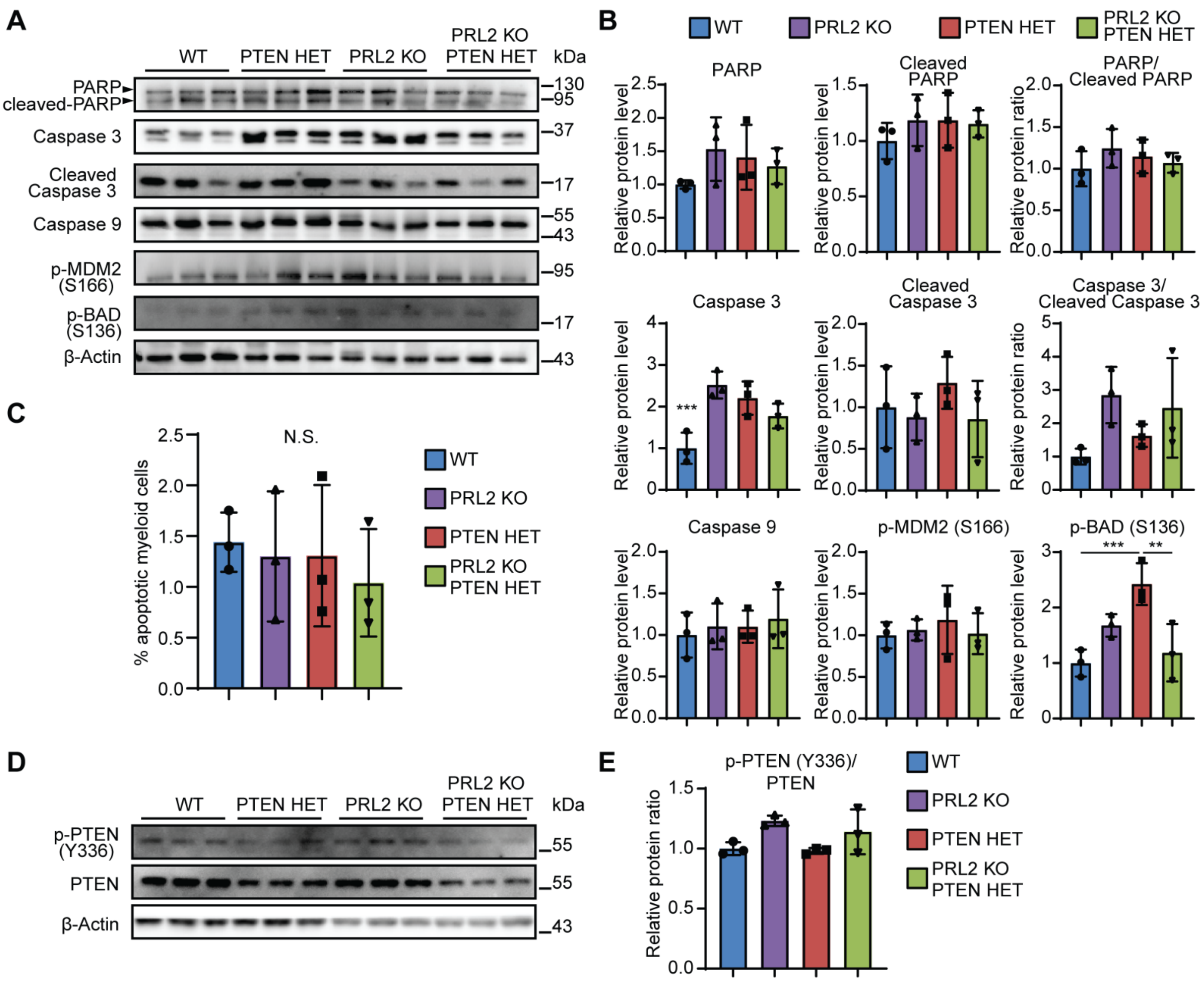

Supplemental Figure 4

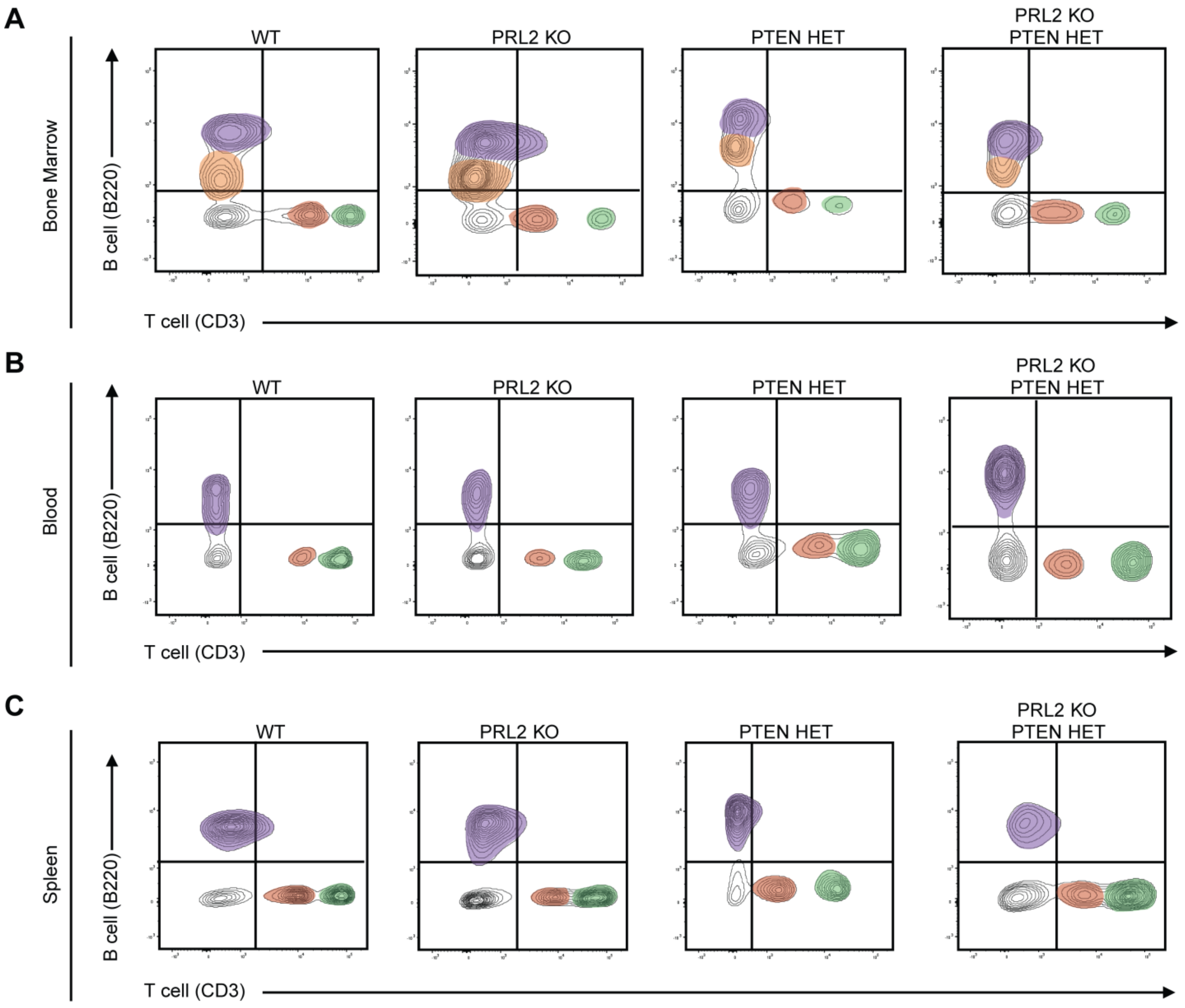

Supplemental Figure 5

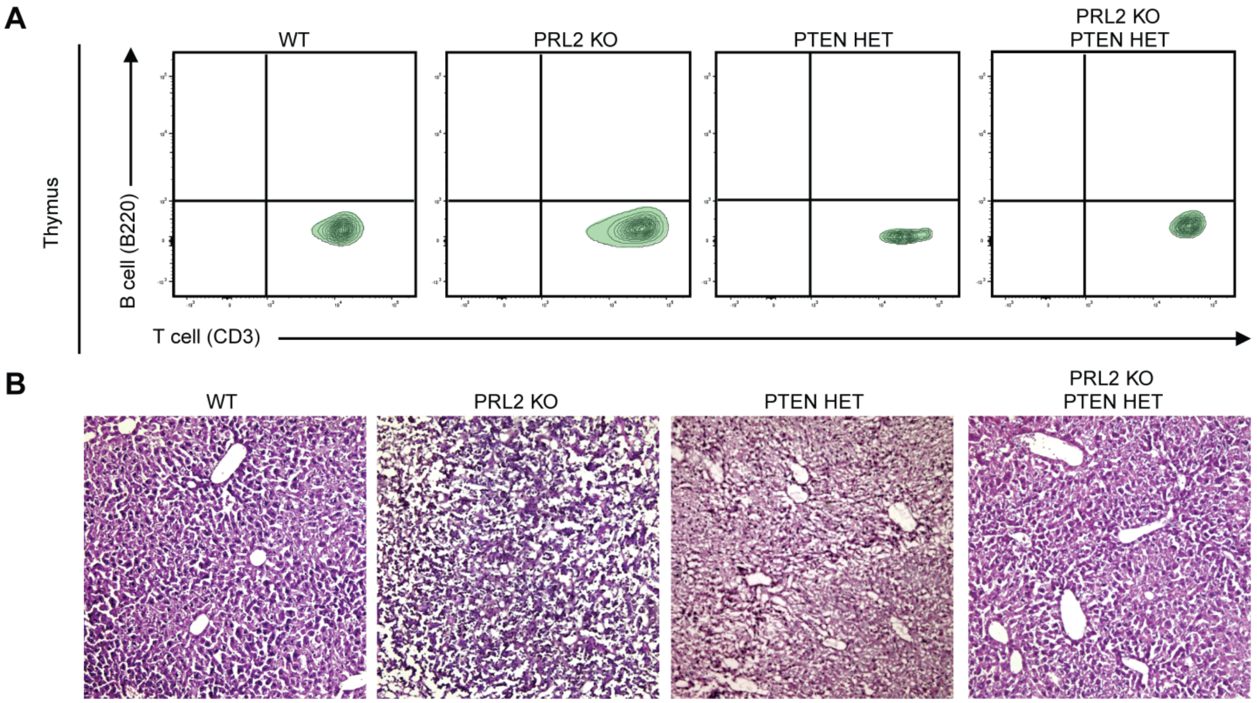

Supplemental Table 1

	WT	PRL2 KO	PTEN HET	PRL2 KO PTEN HET
Splenomegaly	13%	0	22%	9%
Lymphadenopathy	9%	0	31% *	15%
AML	4%	0	56% ***	6%
ALL	0	0	10%	27% **
Skin Cancer	0	0	12%	0
Lung cancer	0	0	0	0
Liver cancer	0	0	0	9%
Prostate cancer	0	0	2%	0
Cervical cancer	4%	0	3%	0
Other cancer	0	0	3%	0

Supplemental Table 2

	WT	PRL2 KO	PTEN HET	PRL2 KO PTEN HET
Splenomegaly	13%	11%	39% *	39% *
Lymphadenopathy	9%	22%	63% ***	64% ***
AML	4%	0%	88% ***	39% ***
ALL	0	0	14% *	30% **
Skin Cancer	0	11%	20% **	15% *
Lung cancer	0	0	3%	0
Liver cancer	0	11%	2%	30% **
Prostate cancer	0	22% *	3%	6%
Cervical cancer	4%	0	7%	9%
Other cancer	0	0	6%	9%
